# E3 ubiquitin ligase ZBTB25 suppresses beta coronavirus infection through ubiquitination of the main viral protease MPro

**DOI:** 10.1016/j.jbc.2023.105388

**Published:** 2023-10-27

**Authors:** Travis B. Lear, Áine N. Boudreau, Karina C. Lockwood, Elise Chu, Daniel P. Camarco, Qing Cao, Matthew Nguyen, John W. Evankovich, Toren Finkel, Yuan Liu, Bill B. Chen

**Affiliations:** 1 Aging Institute, University of Pittsburgh/UPMC, Pittsburgh, Pennsylvania, USA; 2 Vascular Medicine Institute, University of Pittsburgh, Pittsburgh, Pennsylvania, USA; 3 Department of Environmental and Occupational Health, School of Public Health, University of Pittsburgh, Pittsburgh, Pennsylvania, USA; 4 Division of Pulmonary, Allergy and Critical Care Medicine, Department of Medicine, Acute Lung Injury Center of Excellence, University of Pittsburgh, Pittsburgh, Pennsylvania, USA

**Keywords:** SARS-CoV-2, Mpro/NSP5/3CLpro, E3 ubiquitin ligase, protein ubiquitination, host defense

## Abstract

The main protease of severe acute respiratory syndrome coronavirus 2, Mpro, is a key viral protein essential for viral infection and replication. Mpro has been the target of many pharmacological efforts; however, the host-specific regulation of Mpro protein remains unclear. Here, we report the ubiquitin–proteasome-dependent degradation of Mpro protein in human cells, facilitated by the human E3 ubiquitin ligase ZBTB25. We demonstrate that Mpro has a short half-life that is prolonged *via* proteasomal inhibition, with its Lys-100 residue serving as a potential ubiquitin acceptor. Using *in vitro* binding assays, we observed ZBTB25 and Mpro bind to each other *in vitro*, and using progressive deletional mapping, we further uncovered the required domains for this interaction. Finally, we used an orthologous beta-coronavirus infection model and observed that genetic ablation of ZBTB25 resulted in a more highly infective virus, an effect lost upon reconstitution of ZBTB25 to deleted cells. In conclusion, these data suggest a new mechanism of Mpro protein regulation as well as identify ZBTB25 as an anticoronaviral E3 ubiquitin ligase.

The coronavirus disease 2019 pandemic, driven by the β-coronavirus severe acute respiratory syndrome coronavirus 2 (SARS-CoV-2), continues to be a major public health concern. An unprecedented scientific effort has yielded insight into the mechanism of SARS-CoV-2 and its interactions with host cell proteins (1, 2, 3). Key to replication is the viral protein Mpro, also known as NSP5 or 3CL-protease. Mpro separates the nonstructural proteins from the massive polyprotein (pp1a/b), resulting in functional components for complexes, for example, the RNA-dependent RNA polymerase (4). Given its critical role, Mpro has been a therapeutic target, with several inhibitors identified, most notably nirmatrelvir, known as Paxlovid (5, 6, 7, 8). These direct small-molecule inhibitors have demonstrated clinical efficacy.

Foundational studies have demonstrated several viral proteins interact with human proteins, leading to antiviral signaling inactivation, post-translational modification, and inhibition of synthesis to support virulence (9, 10, 11). Of note, viral proteins directly interact with members of the ubiquitin–proteasome system (UPS) (1). The UPS is the major cellular mechanism regulating protein degradation. In this process, a target protein is decorated with polyubiquitin chains, which are recognized by the proteasome to begin digestion (12). An elegant enzymatic cascade of ubiquitin shuttling facilitates substrate ubiquitination, culminating in target binding by E3 ubiquitin ligases (13). The UPS has an evolutionarily conserved antivirus defense role, including the direct degradation of viral proteins (14, 15, 16, 17, 18, 19, 20, 21).

While some studies have explored the stability of viral proteins, the mechanism by which Mpro is proteolytically regulated by the cell remains unclear. Here, we report the results of an unbiased RNAi screen that identified the E3 ubiquitin ligase ZBTB25 as a regulator of Mpro stability and ubiquitination. ZBTB25 directly binds SARS-CoV-2 Mpro, leading to polyubiquitination at its Lys-100 site. Moreover, ZBTB25 demonstrates regulation of the evolutionarily conserved orthologous OC43 Mpro, which upon CRISPR–Cas9 deletion of ZBTB25 leads to increased OC43 virulence *in vitro*. These findings demonstrate a new mechanism of host-based Mpro regulation and identify ZBTB25 as a potential target to augment endogenous antiviral defenses.

## Results

**Figure 1:**
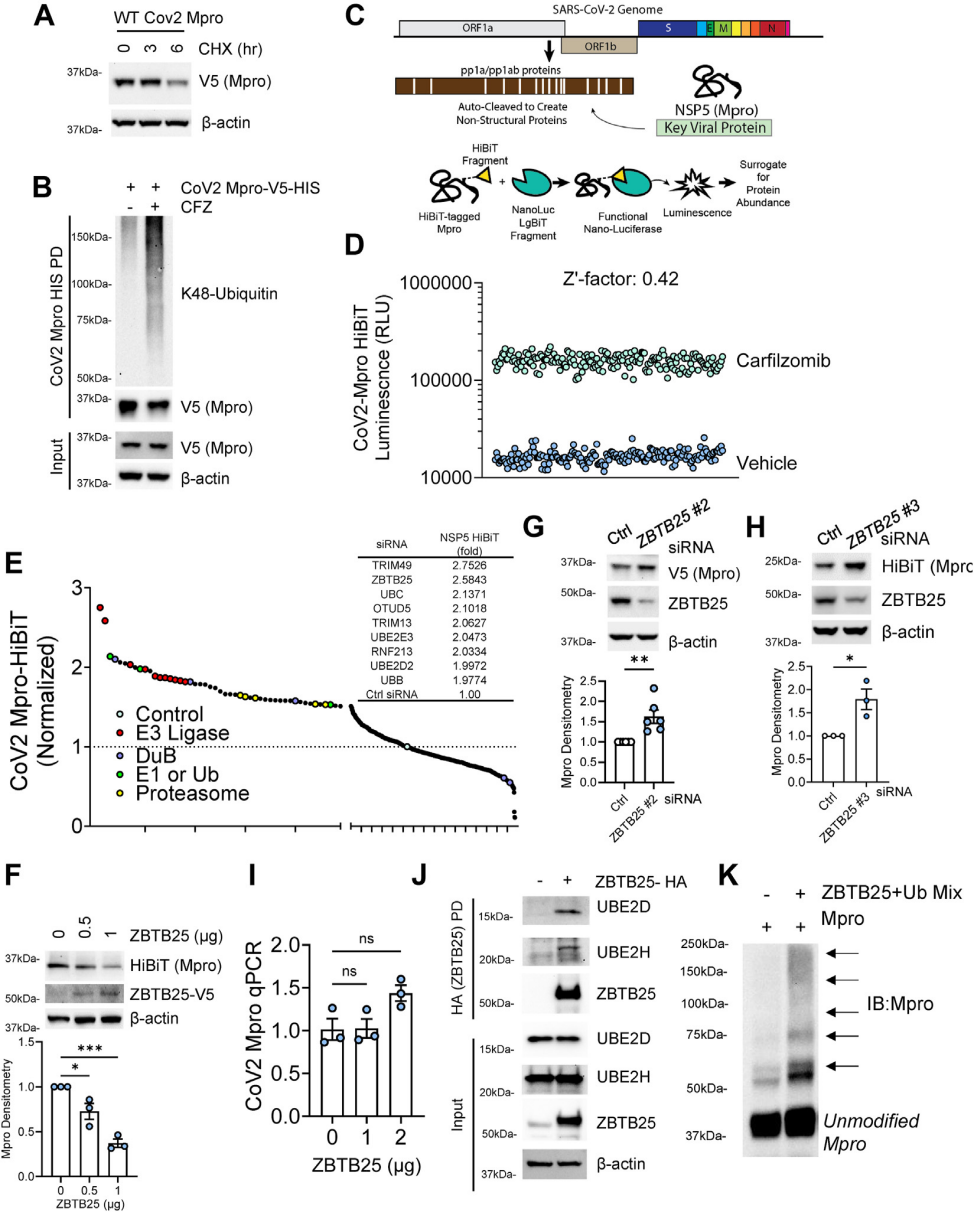
**SARS-CoV-2 Mpro is unstable and regulated by the E3 ubiquitin ligase ZBTB25.***A*, immunoblot analysis of lysate from BEAS-2B cells expressing Mpro and treated with CHX. *B*, immunoblotting of Lys48-linked polyubiquitin from Mpro pulldown from BEAS-2B cells expressing Mpro-V5-HIS following treatment with vehicle or CFZ. *C*, schematic of the role of Mpro in the SARS-CoV-2 proteome and the HiBiT split luciferase system for measuring Mpro stability. *D*, validation of MPro-HiBiT-BEAS2B stable cell line for screening, raw luminescent signal shown, n = 187 per group. *E*, screening of CoV2-Mpro-HiBiT-BEAS-2B stable cell line with RNAi library targeting components of the ubiquitin–proteasome system (∼836 targets). Top hits listed in chart. *F*, immunoblot analysis of CoV2-MPro-HiBiT-BEAS-2B cells with increasing expression of hit ZBTB25. Densitometry below, data are mean ± SEM (n = 3). *G*–*H*, knockdown of ZBTB25 in Mpro-V5-BEAS-2B cells. Densitometry below, data are mean ± SEM (n = 3–6). *I*, quantitative PCR analysis of CoV-2 MPro cells with expression of ZBTB25. Data are mean ± SEM (n = 3). *J*, immunoblotting of ZBTB25 pulldown for coprecipitation of E2-conjugating enzymes. *K*, *in vitro* ubiquitination assay of Mpro by ZBTB25. ∗∗*p* < 0.01; *p* > 0.05, NS; compared with vehicle or control or as indicated by two-sided unpaired *t* test (*G* and *H*) or by one-way ANOVA with Dunnett’s multiple comparisons (*F*–*I*). CFZ, carfilzomib; CHX, cycloheximide; SARS-CoV-2, severe acute respiratory syndrome coronavirus 2.

We expressed CoV-2 Mpro in human airway epithelial BEAS-2B cells and treated with a time course of cycloheximide (CHX). Mpro protein decreased with increasing CHX treatment length (Fig. 1*A*), suggesting a short protein half-life. We next assayed ubiquitination of Mpro, by expression and proteasome inhibitor, carfilzomib (CFZ) treatment prior to Mpro pulldown (PD). We observed increased Mpro K48-linked polyubiquitination with CFZ treatment (Fig. 1*B*). From this, we generated codon-optimized Mpro expression plasmids (1) with the nano-luciferase HiBiT tag (Fig. 1*C*). We have previously used this system with success to screen protein stability (22). Following this, we generated a BEAS-2B cell line stably expressing Mpro-HiBiT and treated multiple replicates (n = 187) with vehicle or CFZ to ascertain the signal window for screening. We calculated the model robustness to have a Z′ factor of 0.42 (relative to CFZ), which is sufficient for use in high-throughput screening (Fig. 1*D*).

We screened for regulators of Mpro using an endoribonuclease-prepared siRNA (esiRNA) library targeting components of the UPS. The top screening hits were the putative E3 ubiquitin ligases TRIM49 and ZBTB25 (Fig. 1*E*). Both proteins have RING-like domains, a key feature of several ubiquitin ligases (23). To validate these hits, we expressed ZBTB25 in cells and observed a significant and dose-dependent decrease in Mpro level (Fig. 1*F*). When we overexpressed TRIM49 in Mpro-expressing BEAS-2B cells, we did not observe the predicted NSP5 protein decrease, thus TRIM49 is not an authentic hit (Fig. S1, *A* and *B*). Next, we knocked down ZBTB25 in Mpro-expressing cells, which led to a significant increase in Mpro protein levels (Fig. 1, *G* and *H*). Furthermore, Mpro transcript level showed no significant change in response to ZBTB25 dose, suggesting the effects of ZBTB25 on Mpro occur post-transcriptionally (Fig. 1*I*). To confirm ZBTB25 as having E3 ubiquitin ligase activity, we observed coprecipitation of E2-conjugating enzymes upon ZBTB25 PD (Fig. 1*J*) and the *in vitro* ubiquitination of Mpro when assembled with the full complement of ubiquitination machinery (Fig. 1*K*).

**Figure 2:**
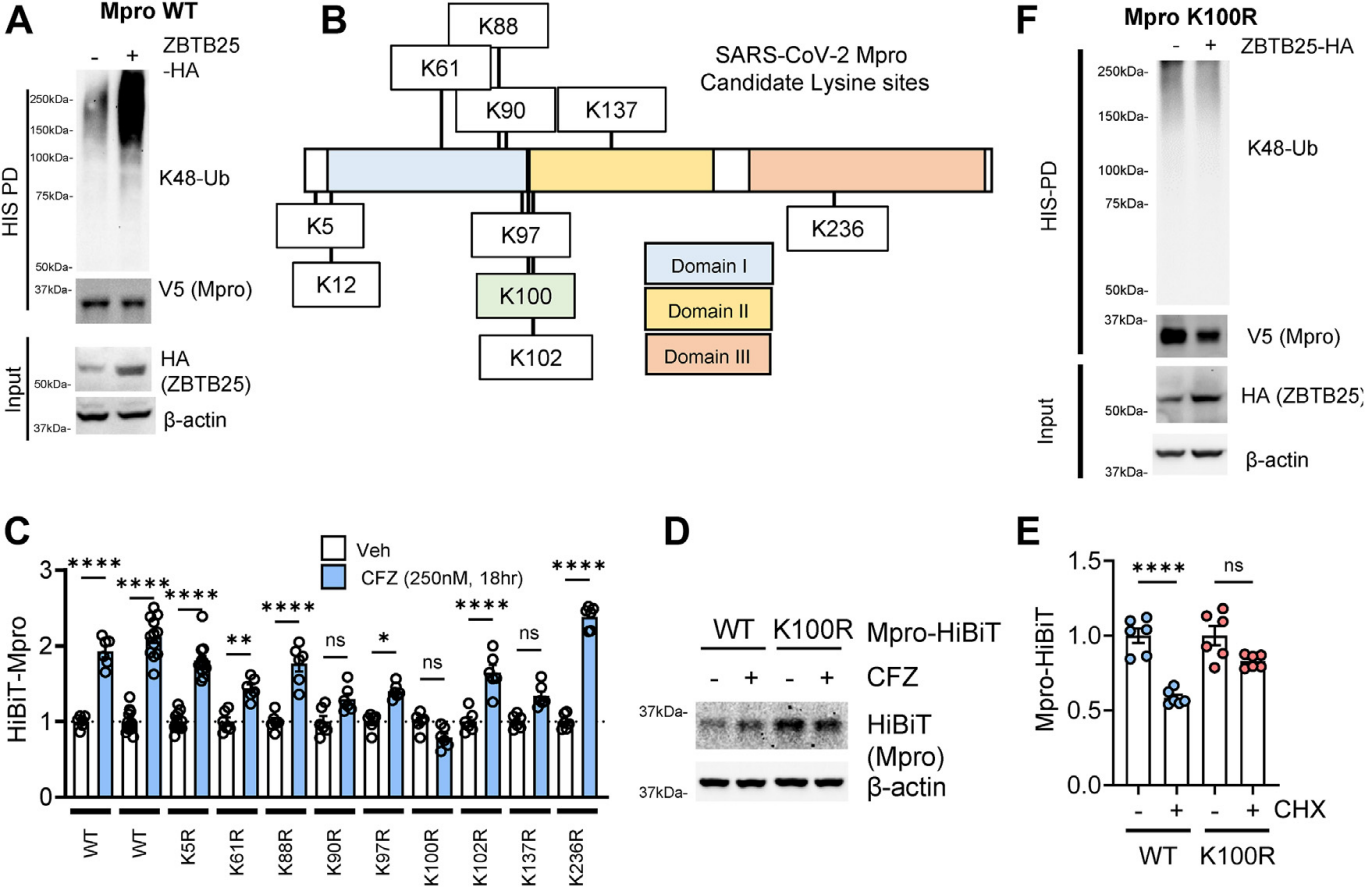
**SARS-CoV-2 Mpro is ubiquitinated by ZBTB25 at its Lys100 site.***A*, cell-based ubiquitination assay of Mpro-WT-V5-HIS with expression of ZBTB25. Mpro was pulled down prior to washing and immunoblotting. *B*, schematic of CoV-2 Mpro protein domains and potential lysine sites for ubiquitin conjugation. *C*, profiling of CoV2 Mpro Lys–Arg (K–R) constructs and their response to proteasomal inhibition, data are a ratio of CFZ-treated luminescence relative to vehicle (Veh)-treated luminescence. Data are mean ± SEM (n = 6–12). *D*, immunoblotting of Mpro-WT-HiBiT and K100R-HiBiT with Veh or CFZ treatment. *E*, luminescent measurement of WT Mpro *versus* K100R during CHX treatment. Data are mean ± SEM (n = 6). *F*, cell-based ubiquitination assay of Mpro-K100R-V5-HIS with expression of ZBTB25. Mpro was pulled down prior to washing and immunoblotting. ∗*p* < 0.05; ∗∗*p* < 0.01; ∗∗∗∗*p* < 0.0001; and *p* > 0.05, NS; compared with Veh or control or as indicated by one-way ANOVA with Tukey’s post hoc test (*C*). CFZ, carfilzomib; CHX, cycloheximide; NS, not significant; CoV-2, severe acute respiratory syndrome coronavirus 2.

To further test the role of ZBTB25 in Mpro regulation, we coexpressed Mpro and ZBTB25 and observed increased Mpro polyubiquitination (Fig. 2*A*). Ubiquitin conjugation proceeds primarily through substrate lysine (K) sites (13). Mpro contains multiple lysine sites, and we mutated several candidates to structurally similar but inactive arginine (R) residues to test the mutants’ stabilities (Fig. 2*B*). HiBiT-tagged WT and mutant Mpro were expressed and treated with CFZ to measure the increase in signal because of proteasomal inhibition. This increase is indicative of the pool of protein undergoing steady-state proteasomal degradation; proteasomal inhibition leads to an increase in the total pool. We hypothesized that mutation of Mpro key lysine sites would impair ubiquitination and subsequent proteasomal degradation, and thus, such structural mutants would not have their levels enhanced by CFZ. While most K–R mutants demonstrated a comparable increase to WT upon proteasomal inhibition, the K-100 mutant (K100R) was impervious to CFZ treatment (Fig. 2*C*). K90R and K137R were also not significantly different; however, they showed a ∼40% increase with CFZ treatment, compared with the complete lack of increase with K100R. Furthermore, immunoblotting showed that CFZ treatment increased the level of Mpro WT but did not affect Mpro K100R (Fig. 2*D*), and Mpro K100R was resistant to CHX-induced protein decrease (Fig. 2*E*). Finally, we coexpressed Mpro K100R and ZBTB25, and in contrast to Figure 2*A*, we observed no difference in polyubiquitination with ZBTB25, further supporting Lys-100 as the predominant residue for Mpro ubiquitination (Fig. 2*F*).

**Figure 3:**
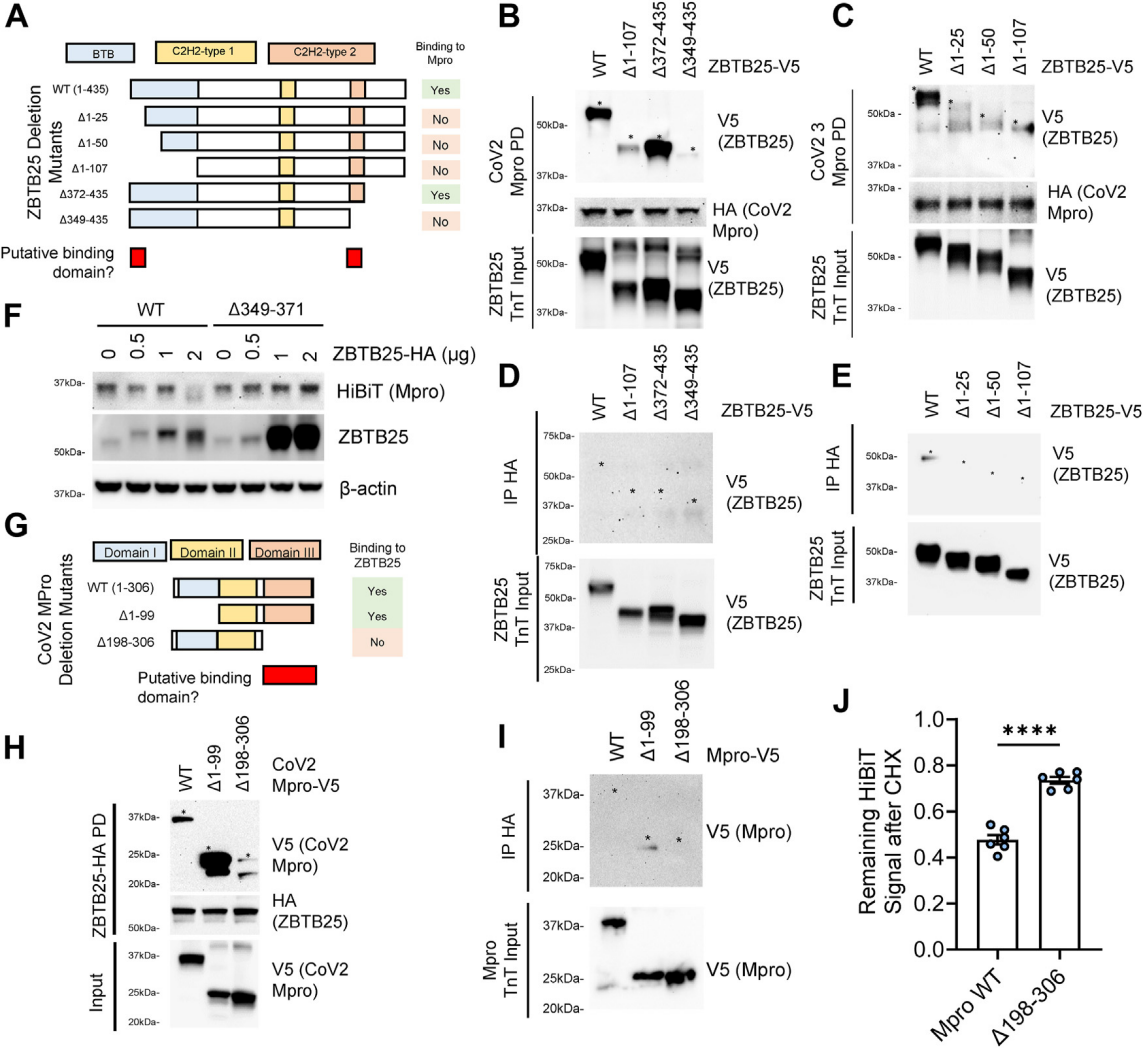
**ZBTB25 and Mpro bind each other through distinct molecular motifs.***A*, schematic of ZBTB25 domains and deletion mapping to find key region for binding CoV2-Mpro. Putative regions essential for Mpro binding are shown in *red*. *B* and *C*, binding assays of ZBTB25 deletion mutants with pulled-down HA-CoV-Mpro. *D* and *E*, ZBTB25 binding assays during control pulldown. *F*, immunoblotting of BEAS-2B-CoV2-Mpro-HiBiT cells expressing ZBTB25 WT or ΔC2H2 #2 dose course. *G*, schematic of CoV2-Mpro domains and deletion mutants for binding assay. *H*, binding assay of Mpro deletion mutants, with pulled-down HA-ZBTB25. *I*, control pulldown with Mpro deletion mutants. *J*, luminescent measurement of WT Mpro *versus* Δ198 to 306 during CHX treatment. Data are mean ± SEM (n = 6). ∗ Indicates bands. CHX, cycloheximide; CoV2, severe acute respiratory syndrome coronavirus 2; HA, hemagglutinin.

**Figure 4:**
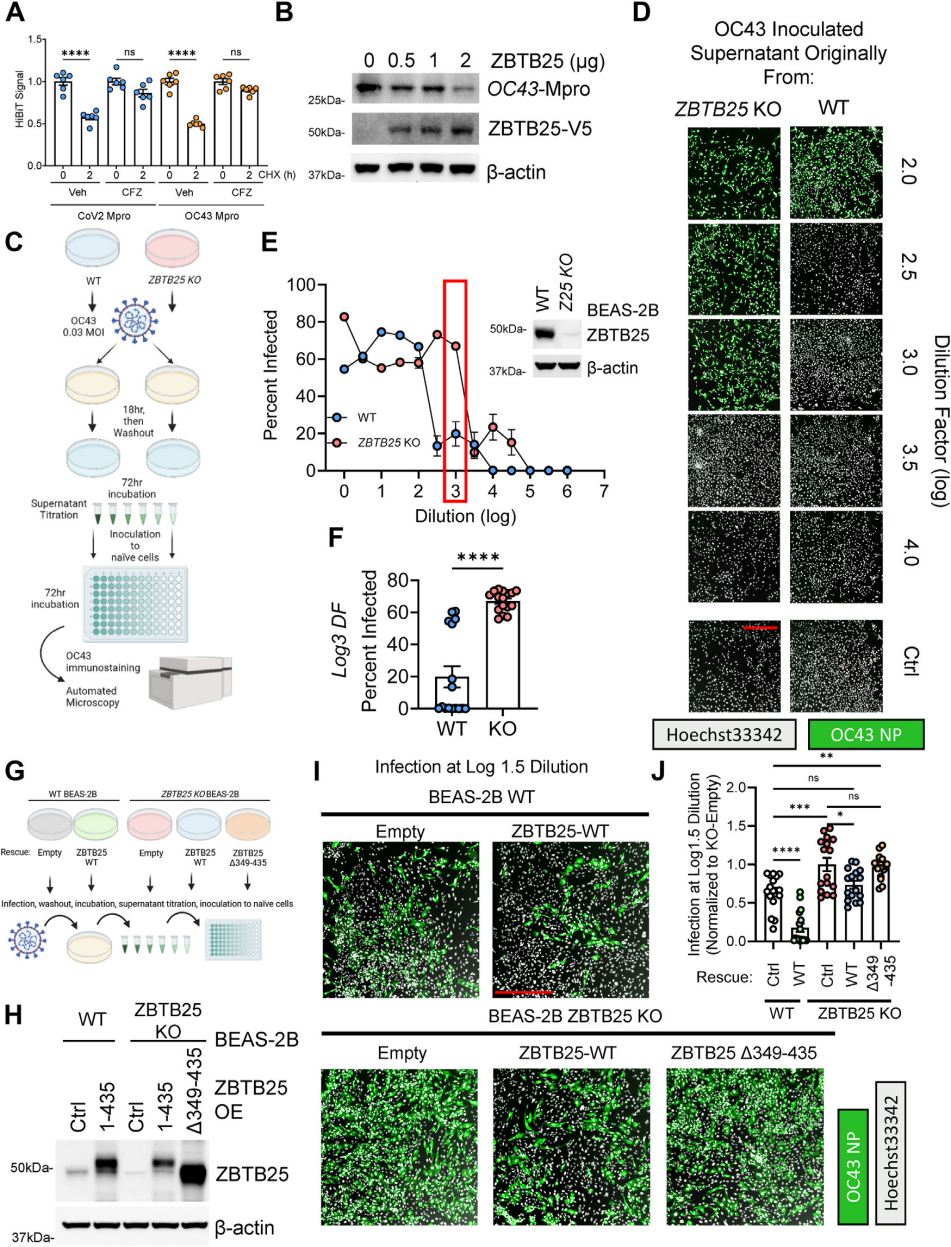
**Depletion of ZBTB25 increases beta-coronavirus infectivity *in vitro*.***A*, CoV2 or OC43 Mpro-HiBiT signal after CHX treatment with or without CFZ. Data are mean ± SEM (n = 6). *B*, immunoblot analysis of BEAS-2B cells expressing OC43-Mpro-HiBiT and an increasing dose of ZBTB25. *C*, treatment schematic for OC43 spreading assay. WT BEAS-2B or ZBTB25 KO cells were inoculated with equal amounts of OC43 before washout and continued incubation. Supernatant was titrated onto naïve BEAS-2B cells, incubated, and processed for immunofluorescent staining of OC43. *D*, representative micrographs of OC43 staining following supernatant titration. Scale bar represents 500 μm. *E* and *F*, quantification of OC43 spreading assay as described in (*C*–*E*). Data are mean ± SEM (n = 16 per dilution/treatment). Immunoblotting of ZBTB25 signal from KO’d cells. *F*, quantification of infected cells proportion from supernatant stemming from WT or ZBTB25 KO at dilution factor “3.” Data are mean ± SEM (n = 16). *G*, reconstitution strategy for ZBTB25 KO cells. *H*, ZBTB25 WT and Δ349 to 435 mutant express well in KO cells. *I*, representative micrographs of OC43 staining following supernatant titration. Scale bar represents 500 μm. *J*, quantification of OC43 spreading from reconstituted cell line supernatant (n = 16 per treatment). NS, *p* > 0.05; ∗*p* < 0.05; ∗∗*p* < 0.01; ∗∗∗*p* < 0.001; and ∗∗∗∗*p* < 0.0001; compared with vehicle or control or as indicated by two-sided unpaired *t* test (*A* and *F*) or as indicated by one-way ANOVA with Tukey’s post hoc test (*J*). CFZ, carfilzomib; CHX, cycloheximide; CoV2, severe acute respiratory syndrome coronavirus 2; NS, not significant.

We next sought to uncover the mechanism through which ZBTB25 and Mpro interact. First, we created deletion mutants of ZBTB25, progressively mapping from either the N- or C-terminal ends (Fig. 3*A*). Proteins were generated *via in vitro* transcription and translation (Promega) and incubated with Mpro WT pulled down from stably expressing cells. The initial assay showed that the BTB domain and the second C2H2 domain are required to bind Mpro (Fig. 3*B*). Deletion of smaller portions of the N terminus still resulted in abrogation of binding, indicating that the first few amino acids of the protein also play a crucial role in the binding of Mpro (Fig. 3*C*). As a control, we noticed no deletion mutant binding when PD occurred without the bait target (*e.g.*, beads-only control) (Fig. 3, *D* and *E*). RING-family E3 ubiquitin ligases utilize their zinc-binding domains for effective ubiquitin conjugation (23). We observed that deletion of the second C2H2 domain abrogated the ability of ZBTB25 function to decrease Mpro (Fig. 3*F*), ostensibly through the loss of a key Mpro-interacting domain. In conjugate, we designed deletion mutants for Mpro, lacking either domain I or domain III (24) (Fig. 4*G*). ZBTB25-HA and Mpro-V5 mutants were generated *in vitro*, and following ZBTB25 PD, Mpro proteins were incubated with ZBTB25 prior to PD and washing. Following elution and immunoblotting, we observed that binding of Mpro and ZBTB25 is lost with the deletion of Mpro domain III (Fig. 4*H*), without nonspecific binding to the PD (Fig. 3*I*). Finally, the Mpro binding mutant displayed resistance to CHX-induced protein loss, potentially because of the loss of the ZBTB25 binding domain (Fig. 3*J*).

Finally, we investigated the biological effect of ZBTB25. We modeled SARS-CoV-2 infection using OC43, a beta coronavirus orthologous to SARS-CoV-2 but with a lower biosafety level. The OC43 Mpro (YP_009555250.1) shows 48% sequence identity to that of CoV-2 (YP_009725301.1), and we note that both proteins show similar stability and regulation by the proteasome during CHX chase (Fig. 4*A*). Furthermore, ZBTB25 similarly affects OC43 Mpro levels (Fig. 4*B*). After this validation, we carried out a viral spreading assay. WT and ZBTB25 KO cells were inoculated with OC43 at 0.03 multiplicity of infection (MOI) for a brief period, and then cells were gently washed and allowed to produce new virus. Supernatant was then titrated to naïve WT cells and processed to measure the *de novo* viral titer (Fig. 4*C*). Naïve cells treated with supernatant from ZBTB25 KO cells showed a higher proportion of infection at more dilute conditions than naïve cells treated with supernatant from WT cells (Fig. 4, *D* and *E*). This is particularly clear around a dilution factor of log3, where approximately 20% of cells treated with WT supernatant were infected, compared with 60% infection in cells treated with ZBTB25 KO supernatant (Fig. 4, *E* and *F*). To validate the effects of the KO cells, we conducted a reconstitution, re-expressing ZBTB25 WT and the binding-deficient mutant to ZBTB25 KO cells prior to conducting the viral spreading assay (Fig. 4*G*). We observed successful re-expression in the KO cells (Fig. 4*H*), and a differential spreading capability from each rescue’s supernatant (Fig. 4, *I* and *J*). There was a significant increase in spreading from KO-Empty relative to WT-Empty, as previously shown. However, re-expression of WT ZBTB25 to KO cells resulted in reduced spreading, resensitizing the cells. This effect was lost with the re-expression of binding-deficient ZBTB25. In addition, overexpression of ZBTB25 WT to WT-BEAS-2B cells resulted in a protective effect relative to baseline expression, further supporting our model. These results demonstrate a role for ZBTB25 in host antiviral defense, in the context of coronavirus infection.

## Discussion

These data demonstrate that SARS-CoV-2 Mpro protein stability is regulated by the host E3 ubiquitin ligase ZBTB25. Furthermore, ZBTB25 modulates the orthologous coronavirus OC43 Mpro protein, and ZBTB25 deletion results in increased viral infectivity. Previous studies of ZBTB25 have noted its role in DNA binding and epigenetic reading (25, 26, 27). In this study, we demonstrate that ZBTB25 may also function as an E3 ubiquitin ligase. Deletion of its key C2H2 domain results in loss of effect in destabilizing SARS-CoV-2 Mpro, presumably by loss of critical E3 function. We observed that loss of the C-terminal region of ZBTB25 and separately the loss of the N terminus ablated binding with CoV2-Mpro protein. Some studies have suggested that ZBTB25 forms a dimer complex (26, 28). In such cases, reciprocal ends of the protein may be required for full complex formation and subsequent binding. Furthermore, Mpro has been noted to homodimerize for its activity (29); the exact stoichiometric configuration of ZBTB25 and Mpro binding will remain an area of study.

Mpro is the focus of many small-molecule programs, with notable clinical success. However, a vulnerability of viral-directed therapies is the mutation of target regions leading to loss of efficacy. Most notable have been mutations in the Spike protein correlating with reduced immunogenicity conferred by anti-SARS-CoV-2 vaccines. However, therapies targeted at other viral proteins such as Mpro have the same risk. Indeed, studies have shown that natural mutations in Mpro render nirmatrelvir ineffective, and that these mutations are already circulating in the population (30). Future studies will investigate the possibility of targeting ZBTB25. These efforts fall under the greater paradigm of identifying host-specific targets as avenues for antiviral efforts. Our group and others have shown that modulation of specific host targets can oppose viral infectivity, including targets such as the human receptor proteins for Ace2 (31), and adapter TMPRSS2 (22, 32), antiviral cytokine signaling (33), or upregulation of lysosomal activity through TFEB stabilization (34). By uncovering components of the UPS that regulate viral proteins, we add new host-centric avenues for intervention and potential therapeutic targeting for current and future pandemics.

## Experimental procedures

### Materials

SARS-CoV-2 Mpro complementary DNA (cDNA) sequence (pLVX-EF1alpha-SARS-CoV-2-nsp5-2xStrep-IRES-Puro) was a gift from Nevan Krogan (1) (Addgene plasmid #141370). High-Capacity RNA-to-cDNA Kit (catalog no.: 4387406) and PowerUp SYBR Green Master Mix (catalog no.: A25741) were from Applied Biosystems. BEAS-2B (catalog no.: CRL-9609) and HCT-8 (catalog no.: CCL-244) were from American Type Culture Collection (ATCC). CFZ (catalog no.: 17554) was from Cayman Chemical. Anti-K48 ubiquitin (D9D5) (catalog no.: 8081) was from Cell Signaling Technology. OC43 Mpro cDNA sequence (catalog no.: HcCD00960233) was from DNASU. Ubiquitinylation kit (catalog no.: BML-UW9920-0001) was from Enzo. Dulbecco's modified Eagle's medium/F-12 (catalog no.: 11320082), EMEM (catalog no.: 670086), fetal bovine serum (catalog no.: 26140079), and Opti-MEM (catalog no.: 31985062) were from Gibco. ZBTB25 (catalog no.: TOLH-1509863) and Trim49 (catalog no.: TOLH-1427062) cDNA were from CCSB-Broad (35) (Horizon Discovery). Anti-OC43, clone 541-8F (catalog no.: MAB9012) was from MilliporeSigma. Phusion polymerase (catalog no.: M0530), Quick CIP (catalog no.: M0525), and Quick Ligation Kit (catalog no.: M2200) were from New England Biolabs. Lullaby RNAi transfection reagent (catalog no.: LL70500) was from Oz Biosciences. TnT Transcription/Translation kit (catalog no.: L1170), Nano-Glo HiBiT Lytic (catalog no.: N3030), CellTiter-Glo (catalog no.: G9241), Nano-Glo HiBiT Blotting (catalog no.: N2410), pFC37K-HiBiT plasmid (catalog no.: N2391) were from Promega. Anti-ZBTB25 (catalog no: 25631-1-AP) and anti-NSP5 (catalog no.: 29286-1-AP; Research Resource Identifier [RRID]: AB_2918269) were from Proteintech. Anti UBE2D (catalog no.: SC-166278; RRID: AB_2210152), Anti-UBE2H (catalog no.: SC-100620; RRID: AB_2210469) were from Santa Cruz Biotechnology. Ubiquitination esiRNA library was from Sigma–Aldrich (36). Recombinant Mpro was from Sinobiological (catalog no.: 40594-V56E). Anti-β-actin (catalog no.: AM4302), anti-rabbit horseradish peroxidase (catalog no.: 31460), anti-mouse horseradish peroxidase (catalog no.: 31430), anti-mouse AlexaFluor 488 (catalog no.: A-11001), hemagglutinin (HA) tag (2-2.2.14) (catalog no.: 26183), V5 tag (catalog no.: R960-25), CHX (catalog no.: J66665-06), Dynabeads His-Tag (catalog no.: 10103D), pcDNA3.1D (catalog no.: K490001), Hoechst 33342 (catalog no.: H3570), Lipofectamine 3000 (catalog no.: L3000015), anti-HA Beads (catalog no.: 88836), Protein A/G Beads (catalog no.: 88802), protease inhibitor (catalog no.: A32963), and TOP10 (catalog no.: C404010) were from Thermo Fisher Scientific. pRK5-HA-KPTN was a gift from David Sabatini (Addgene plasmid #87042; RRID: Addgene_87042 (37)).

### Cell culture

BEAS-2B cells were cultured in HITES media + 10% fetal bovine serum (FBS) and penicillin–streptomycin. HCT-8 cells were cultured in RPMI1640 medium + 10% FBS and penicillin–streptomycin. KO cell lines were prepared using lentivirus as described previously (34). Briefly, sequences for target genes were created with GPP single guide RNA Designer (38) and cloned into pLENTI-CRISPR-vs2 (39). Lentiviral particles were generated by coexpression of single guide RNA–encoded pLENTI-CRISPR-vs2 with psPAX2 and pMD2.G in human embryonic kidney 293T cells. Target cells were incubated with lentivirus prior to antibiotic selection and generation of monoclonal populations. Validation of KO was determined by immunoblotting.

### Viral propagation and inoculation

The propagation methods and times are based on ATCC recommendations.

### Human coronavirus OC43, betacoronavirus 1, ATCC VR-1558, Lot no.: 70034234

OC43 virus was prepared as previously described (34). Briefly, HCT-8 cells were cultured in T75 flasks to 90% confluence, and cells were washed two times with serum-free medium. About 300 μl of the stock OC43 was diluted in 5 ml of serum-free medium, and the virus dilution was adsorbed on the cells for 1 h at 34 °C with 5% CO_2_. The adsorption was ended by adding 10 ml of serum-free medium on the cells, and the virus was propagated for 5 days at 34 °C with 5% CO_2_. The viral supernatant was collected by centrifugation at 1000*g* for 10 min. Larger batches were made similarly.

### Cell inoculation

Compound and virus dilutions were prepared in Dulbecco's modified Eagle's medium low glucose with 2% FBS and penicillin–streptomycin. The calculated MOI of the virus was noted in the experiment.

### Immunoblotting

Protein samples were collected by lysing cells in radioimmunoprecipitation assay buffer (50 mM Tris–HCl, pH 7.6, 150 mM NaCl, 1.0% Triton X-100, 0.1% SDS, and 0.5% sodium deoxycholate) supplemented with protease inhibitor. Following sonication (20% for 12 s), lysate was centrifuged (12,000 xrcf, 10 min, 4 °C). Protein concentration was determined, and normalized samples were prepared in reducing Laemmli buffer (final concentration: 50 mM Tris–HCl [pH 6.8], 2% SDS, 10% glycerol, and 100 mM DTT). 1° antibodies were used at 1:1000 dilution, and 2° antibodies were used at 1:5000 dilution. Blots were developed using West Femto Maximum Sensitivity Substrate on UVP GelStudio Series imager (Analytik-Jena). Please see uncropped blots in the supporting information.

### Protein stability assays

Protein half-life was measured by time-dependent treatment of CHX (0.1 mg/ml) prior to cell collection and immunoblotting. Response to proteasomal inhibition was measured by CFZ treatment (1 μM) for 18 h or as indicated before collection for PD, immunoblot analysis, or lytic HiBiT detection following the manufacturer’s protocol.

### UPS RNAi screening assay

RNAi screening of SARS-CoV-2 Mpro-HiBiT-expressing BEAS-2B cells with library targeting components of the ubiquitination pathway was conducted similar to previous description (36). Briefly, 0.2 μg of esiRNA (Sigma) was diluted with Opti-MEM and mixed with Lullaby reagent for 20 min before mixture with BEAS-2B cells stably expressing CoV-2 Mpro-HiBiT to a final density of 3e3 cells per well. After 72 h of silencing, cells were analyzed with Nano-Glo HiBiT Lytic Detection System and CellTiter-Glo 2.0 Cell Viability Assay. Luminescent signal was normalized to control siRNA signal.

### Plasmid transfection

HA constructs were subcloned from plasmid originally encoding KPTN-HA (37). Plasmids were transfected using Lipofectamine3000 following the manufacturer’s protocol and analyzed at 18 to 48 h post-transfection. Knockdown was accomplished using Lullaby reagent and protocol and detected at 48 to 72 h postadministration.

### Gene expression assay

Total RNA from BEAS-2B cells stably expressing SARS-CoV-2 Mpro-HiBiT and transfected with ZBTB25-V5 was extracted using RNA extraction kit (Bioland), and cDNA was generated through High Capacity RNA-to-cDNA kit. RNA levels were determined through quantitative PCR using PowerUp SYBR Green on Viia 7 system (Applied Biosystems) and ΔΔCt calculation. The following oligos were used: GAPDH: 5-AAGCTCATTTCCTGGTATGACA-3, 5-TCTTACTCCTTGGAGGCCATGT-3, Mpro-HiBiT: 5-AGTGCAGATTACGTGTCGGG-3, 5-CGTTCCCCAGCGGTAAAGTT-3.

### HIS-tag PD

CoV-2 Mpro WT and mutant sequence in pcDNA3.1D-V5-HIS were expressed in BEAS-2B cells prior to treatment; then cells were lysed and Mpro protein was precipitated with Dynabead HIS-resin (Thermo). Precipitate was eluted in 1× Laemmli buffer at 95 °C for 10 m and resolved through SDS-PAGE immunoblotting.

### Binding assays

Binding assays were conducted as previously described (40). Briefly, SARS-CoV-2 Mpro-HA proteins were immunoprecipitated using 1:100 antibody dilution in immunoprecipitation buffer (50 mM Tris–HCl [pH 7.6], 150 mM NaCl, 0.25% v/v Triton X-100) for 4 h at +4 °C, or ZBTB25-HA was generated *via* TnT expression and captured with anti-HA beads. Following capture, the lysate/bait was incubated in protein A/G agarose resin for an additional 2 h. V5-tagged binding mutants were *in vitro* synthesized using TnT expression kits and allowed to bind overnight. Resin was washed in immunoprecipitation buffer for three rounds of 5 min washes, and protein was eluted in 1× Laemmli buffer at 88 °C for 5 min prior to analysis.

### *In vitro* ubiquitination assays

Assays were performed as previously described (41). Briefly, recombinant Mpro, 50 mM Tris (pH 7.6), 5 mM MgCl_2_, 0.6 mM DTT, 2 mM ATP, 1.5 ng/ml E1, 10 ng/ml ubiquitin E2-conjugating enzymes, 1 mg/ml ubiquitin, and *in vitro* synthesized ZBTB25 were incubated for 90 min at 30 °C prior to analysis by immunoblotting.

### Infectivity assay

WT or *ZBTB25* KO BEAS-2B cells were inoculated with equal amounts of OC43 (0.03 MOI) for 18 h at 34 °C. Following the initial infection, cells were gently but thoroughly washed (three times fresh media) and incubated for an additional 72 h (34 °C). Following the second incubation, equal amounts of media supernatant were removed and titrated onto naïve WT-BEAS-2B cells. Following 72 h incubation, cells were fixed and immunostained for OC43. Cells were imaged with GE InCell2000, and the proportion of infected cells was calculated through CellProfiler (42).

### Quantification and statistical analysis

Statistical comparisons were performed in GraphPad Prism 9 (GraphPad Software, Inc). All statistical details of experiments can be found in the figure legend.

## Data availability

The datasets generated during and/or analyzed during the current study are available from the corresponding author on reasonable request.

## Supporting information

This article contains supporting information.

## Conflict of interest

The authors declare that they have no conflicts of interest with the contents of this article.

## Supporting information

ScreeningData

Supplemental figure

UncroppedBlots-FWMarkers
