# Supplementary material for: E3 ubiquitin ligase ZBTB25 suppresses beta coronavirus infection through ubiquitination of the main viral protease MPro
Source: J Biol Chem. 2023 Oct 27;299(12):105388. doi: 10.1016/j.jbc.2023.105388 (PMC10679490; doi:10.1016/j.jbc.2023.105388)
Supplement: Supplemental figure [file mmc2.docx]

**Supplementary Figure 1: Trim49 is a false hit for Mpro regulation**

**A-B.** Immunoblot analysis of BEAS-2B-CoV2-Mpro-HiBiT (A) or CoV2-MPro-HA (B) with TRIM49 expression. Densitometry below, data are mean±SEM (n=3). NS; compared to vehicle or control or as indicated by one-way ANOVA with Dunnett’s multiple comparisons (A-B).
