# Supplementary figures and images for: E3 ubiquitin ligase ZBTB25 suppresses beta coronavirus infection through ubiquitination of the main viral protease MPro
Source: J Biol Chem. 2023 Oct 27;299(12):105388. doi: 10.1016/j.jbc.2023.105388 (PMC10679490; doi:10.1016/j.jbc.2023.105388)

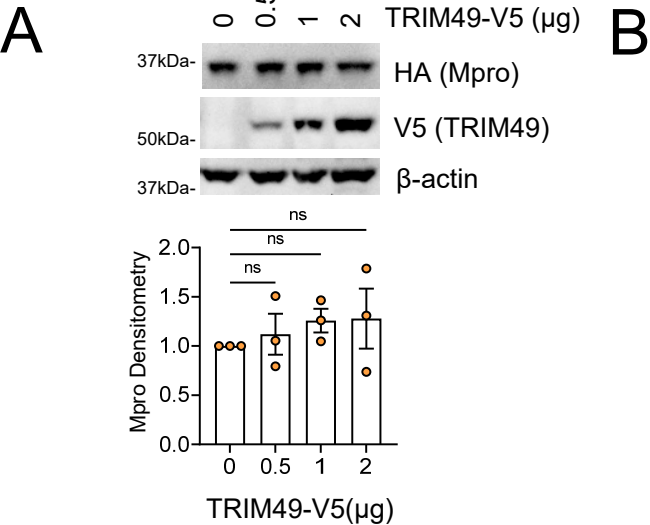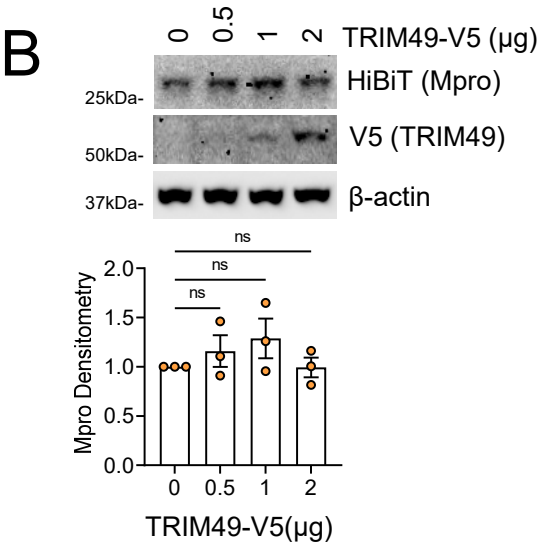

Supplement: UncroppedBlots-FWMarkers [file mmc3.pdf]
