# Supplementary material for: E3 ubiquitin ligase ZBTB25 suppresses beta coronavirus infection through ubiquitination of the main viral protease MPro
Source: J Biol Chem. 2023 Oct 27;299(12):105388. doi: 10.1016/j.jbc.2023.105388 (PMC10679490; doi:10.1016/j.jbc.2023.105388)
Supplement: Figure S1 [file mmc4.pdf]

E3 ubiquitin ligase ZBTB25 suppresses beta coronavirus infection through ubiquitination of the main viral protease MPro

Uncropped Blots

Fig. 1A

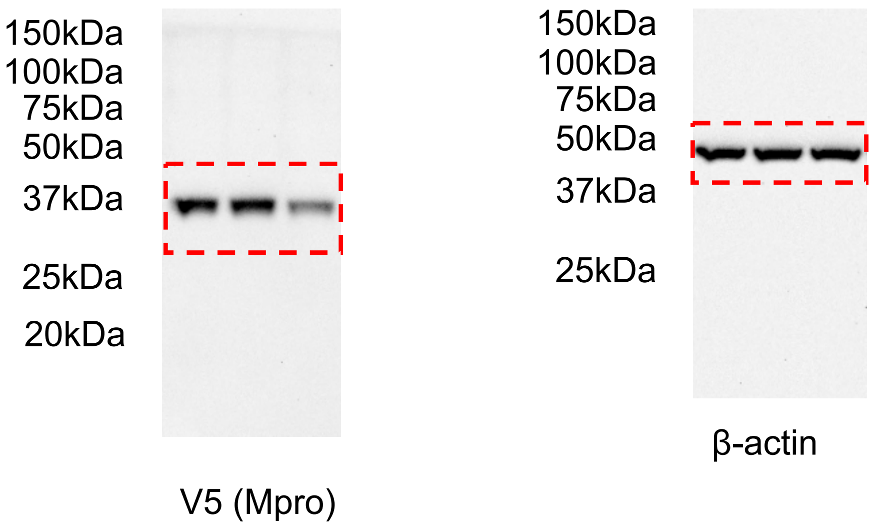
150kDa
100kDa
75kDa
50kDa
37kDa
25kDa
20kDa
V5 (Mpro)
150kDa
100kDa
75kDa
50kDa
37kDa
25kDa
β-actin

Fig. 1B

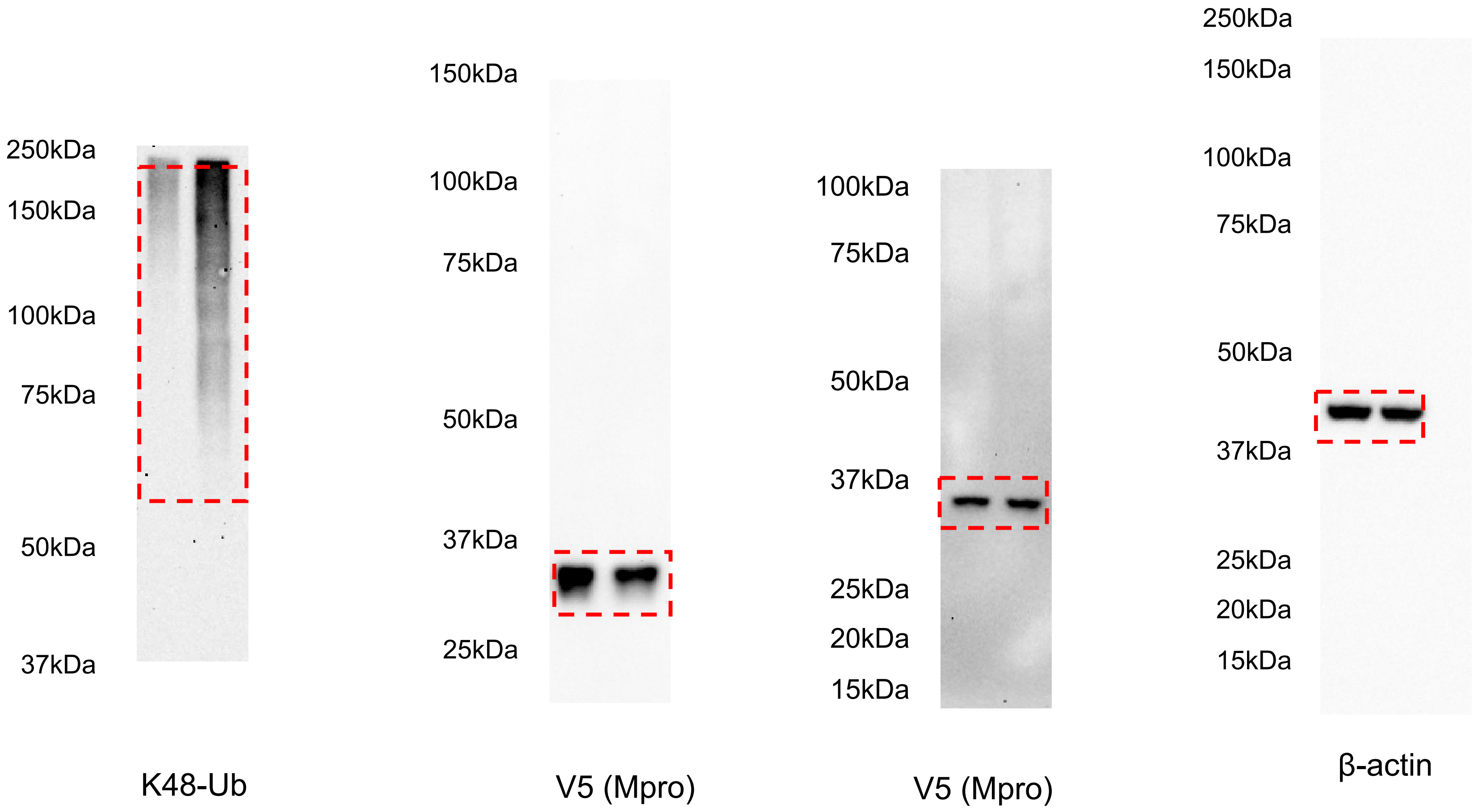
250kDa
150kDa
100kDa
75kDa
50kDa
37kDa
K48-Ub
150kDa
100kDa
75kDa
50kDa
37kDa
25kDa
V5 (Mpro)
100kDa
75kDa
50kDa
37kDa
25kDa
20kDa
15kDa
V5 (Mpro)
250kDa
150kDa
100kDa
75kDa
50kDa
37kDa
25kDa
20kDa
15kDa
β-actin

Fig. 1G

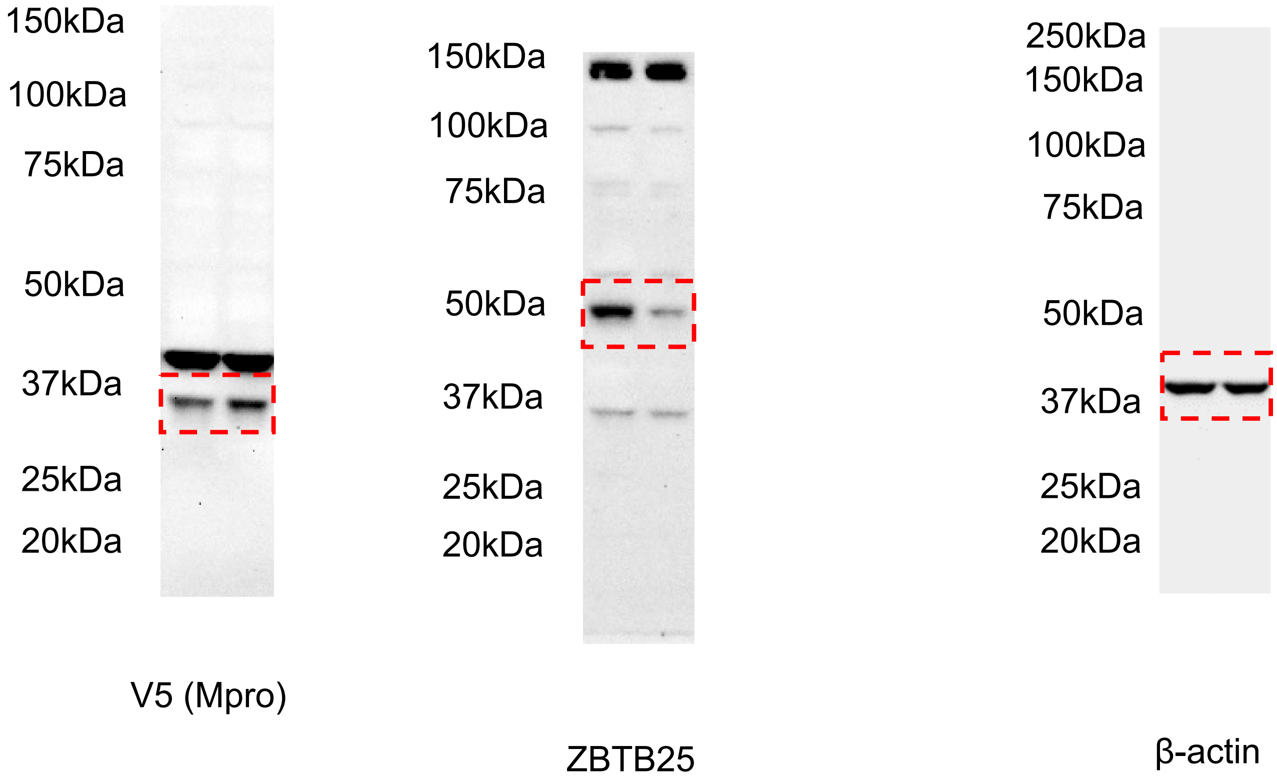
150kDa
100kDa
75kDa
50kDa
37kDa
25kDa
20kDa
V5 (Mpro)
150kDa
100kDa
75kDa
50kDa
37kDa
25kDa
20kDa
ZBTB25
250kDa
150kDa
100kDa
75kDa
50kDa
37kDa
25kDa
20kDa
β-actin

Fig. 1H

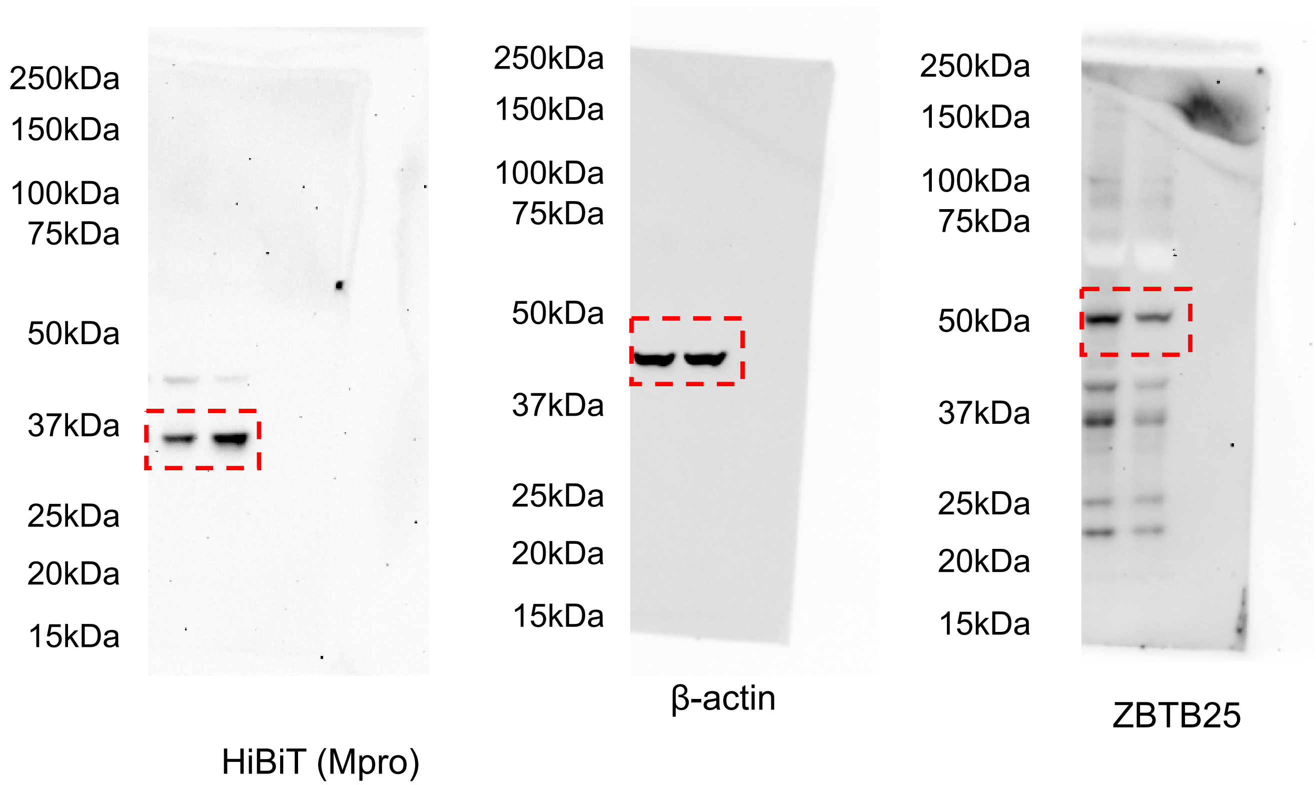
250kDa
150kDa
100kDa
75kDa
50kDa
37kDa
25kDa
20kDa
15kDa
HiBiT (Mpro)
250kDa
150kDa
100kDa
75kDa
50kDa
37kDa
25kDa
20kDa
15kDa
β-actin
250kDa
150kDa
100kDa
75kDa
50kDa
37kDa
25kDa
20kDa
15kDa
ZBTB25

Fig. 1F

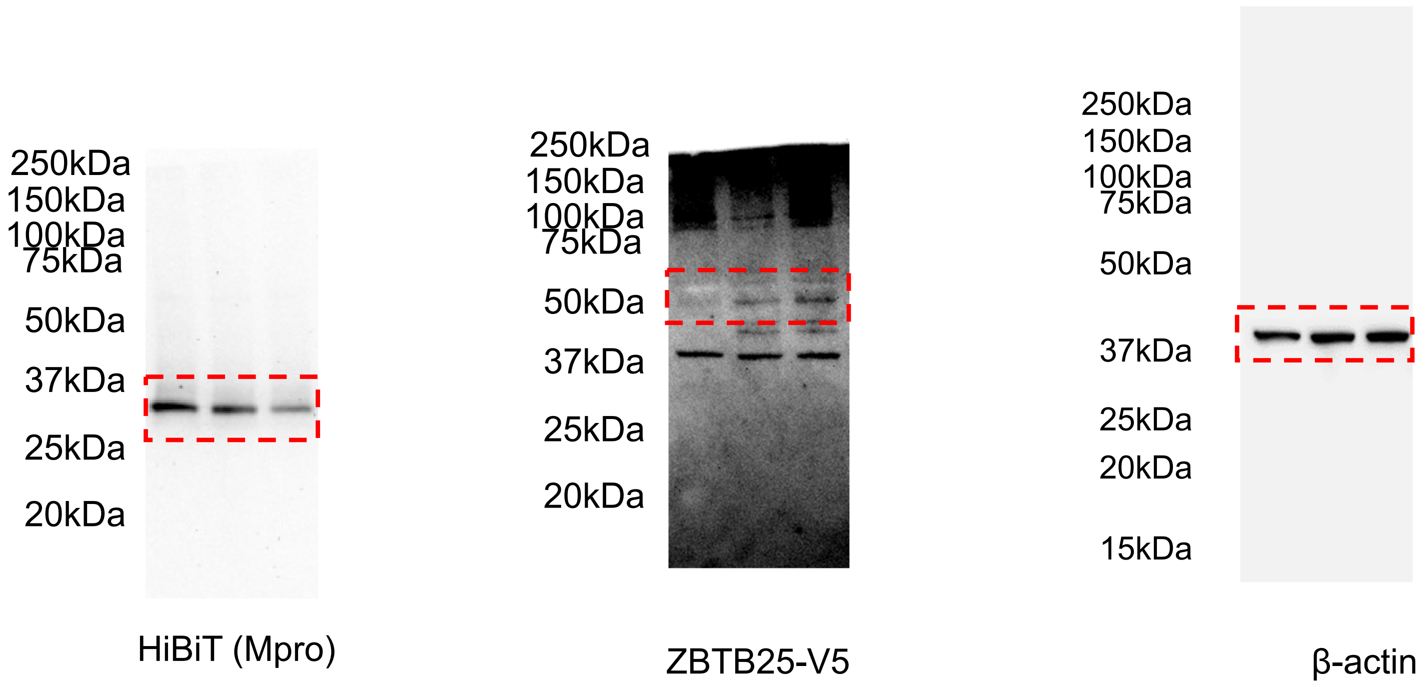
250kDa
150kDa
100kDa
75kDa
50kDa
37kDa
25kDa
20kDa
HiBiT (Mpro)
250kDa
150kDa
100kDa
75kDa
50kDa
37kDa
25kDa
20kDa
ZBTB25-V5
250kDa
150kDa
100kDa
75kDa
50kDa
37kDa
25kDa
20kDa
15kDa
β-actin

Fig. 1J

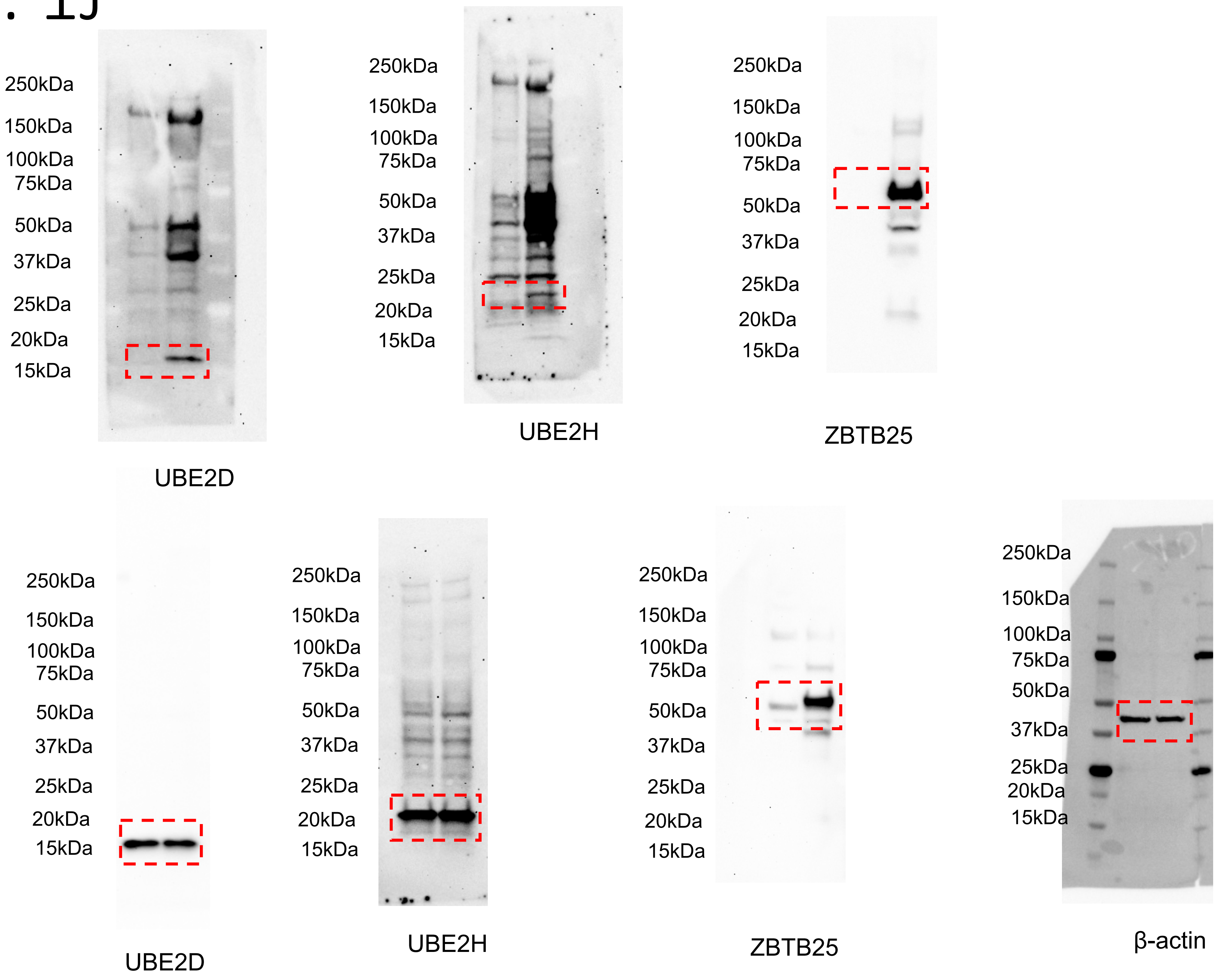
250kDa
150kDa
100kDa
75kDa
50kDa
37kDa
25kDa
20kDa
15kDa
UBE2D
UBE2H
ZBTB25
UBE2D
UBE2H
ZBTB25
β-actin

Fig. 1K

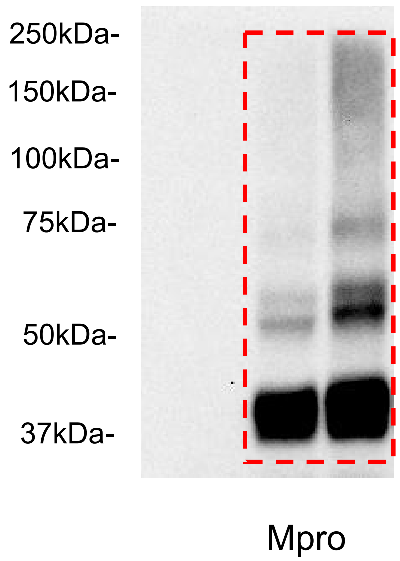

Fig. 2A

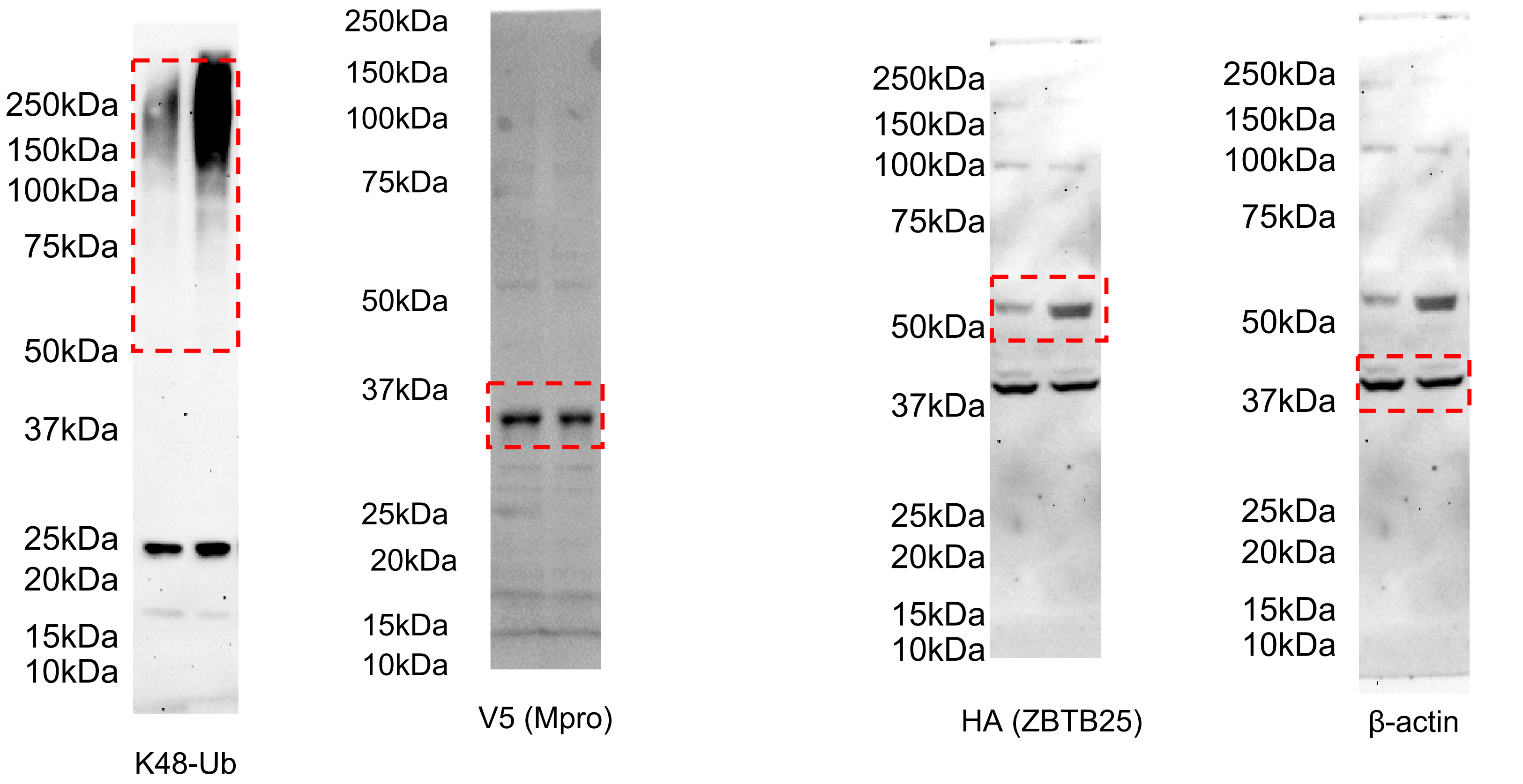

250kDa
150kDa
100kDa
75kDa
50kDa
37kDa
25kDa
20kDa
15kDa
10kDa
K48-Ub
250kDa
150kDa
100kDa
75kDa
50kDa
37kDa
25kDa
20kDa
15kDa
10kDa
V5 (Mpro)
250kDa
150kDa
100kDa
75kDa
50kDa
37kDa
25kDa
20kDa
15kDa
10kDa
HA (ZBTB25)
250kDa
150kDa
100kDa
75kDa
50kDa
37kDa
25kDa
20kDa
15kDa
10kDa
β-actin

Fig. 2D

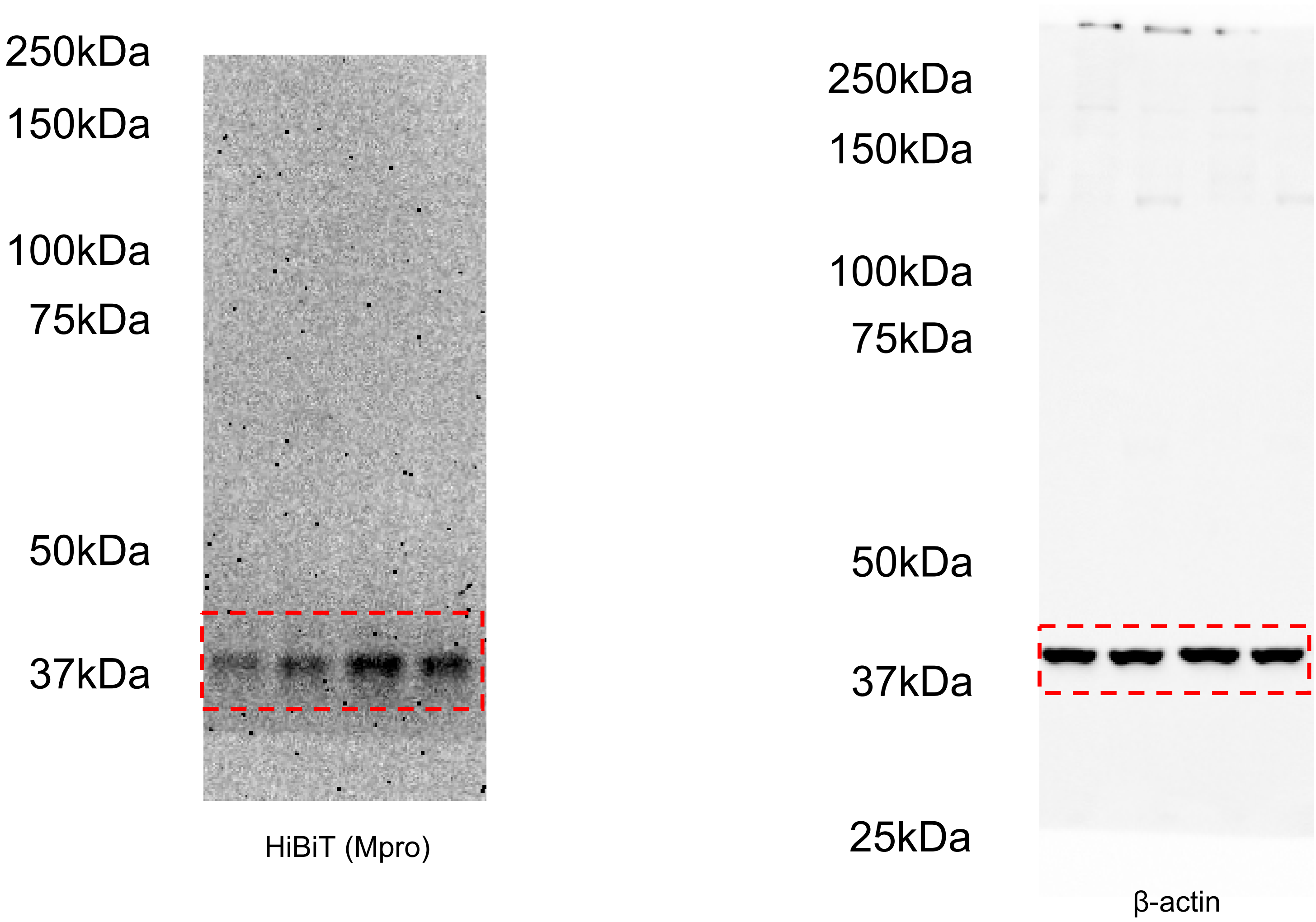
250kDa
150kDa
100kDa
75kDa
50kDa
37kDa
HiBiT (Mpro)
250kDa
150kDa
100kDa
75kDa
50kDa
37kDa
25kDa
β-actin

Fig. 2F

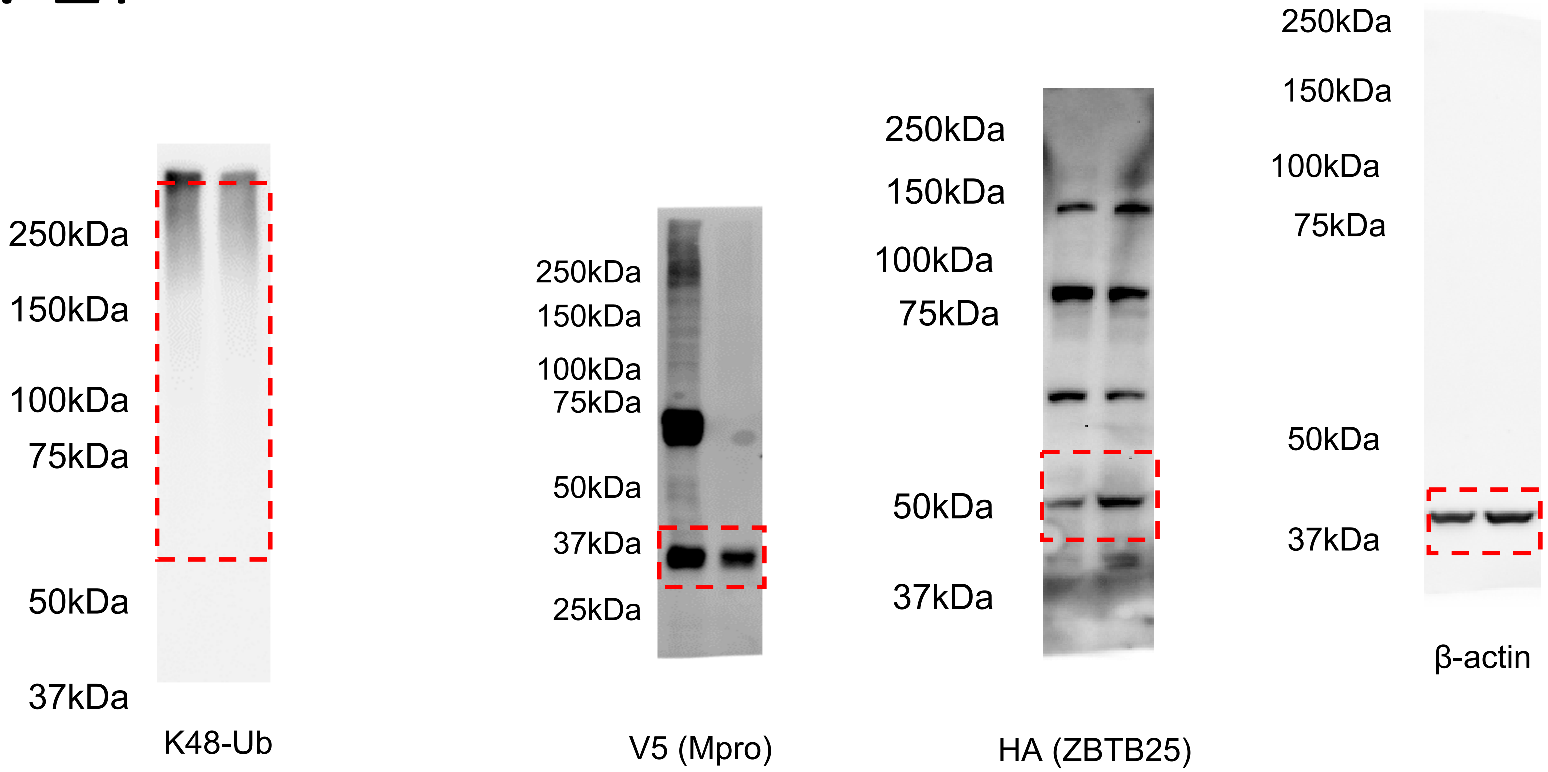
250kDa
150kDa
100kDa
75kDa
50kDa
37kDa
K48-Ub
250kDa
150kDa
100kDa
75kDa
50kDa
37kDa
25kDa
V5 (Mpro)
250kDa
150kDa
100kDa
75kDa
50kDa
37kDa
HA (ZBTB25)
250kDa
150kDa
100kDa
75kDa
50kDa
37kDa
β-actin

Fig. 3B

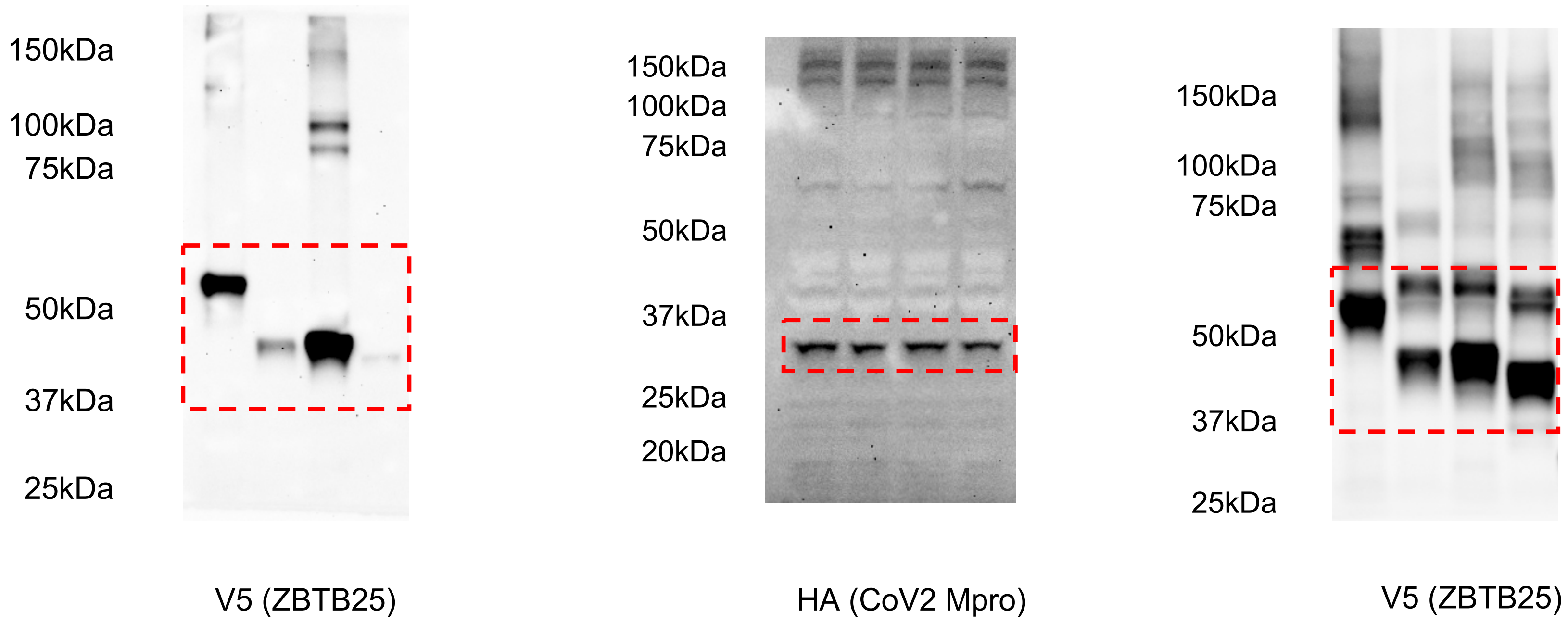
150kDa
100kDa
75kDa
50kDa
37kDa
25kDa
V5 (ZBTB25)
150kDa
100kDa
75kDa
50kDa
37kDa
25kDa
20kDa
HA (CoV2 Mpro)
150kDa
100kDa
75kDa
50kDa
37kDa
25kDa
V5 (ZBTB25)

Fig. 3C

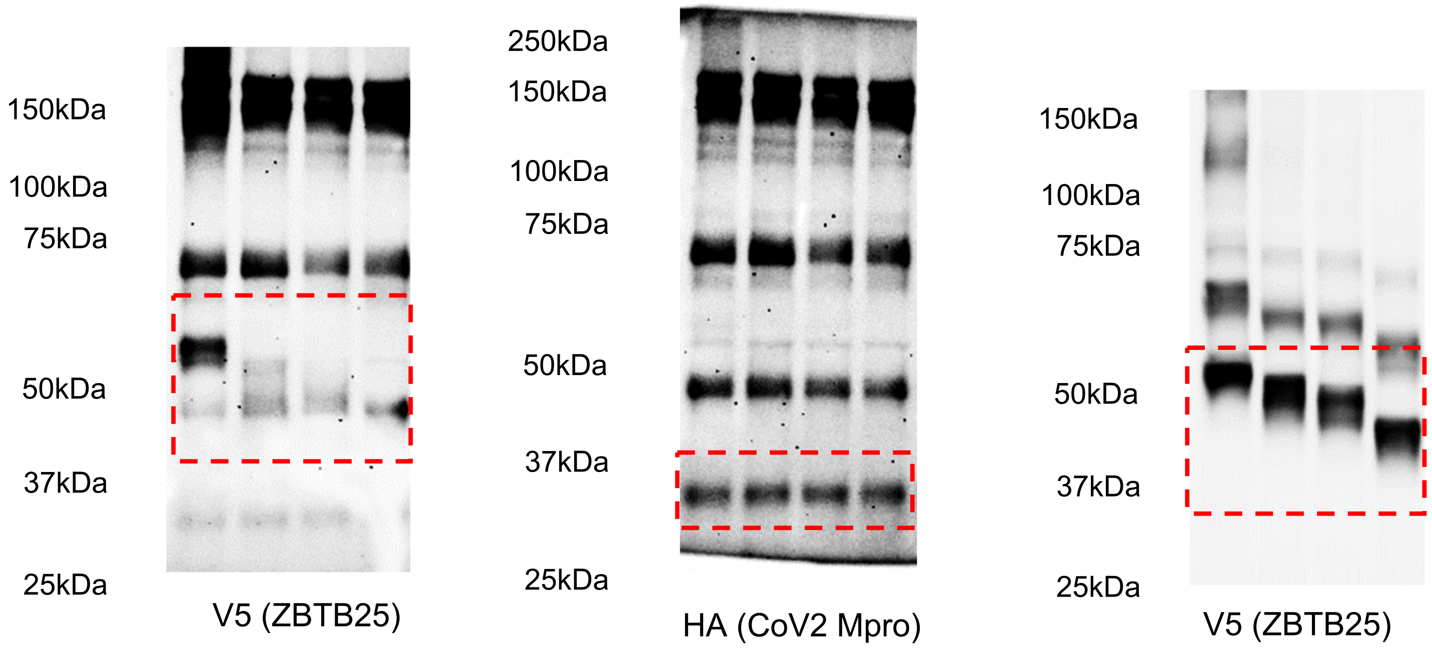
150kDa
100kDa
75kDa
50kDa
37kDa
25kDa
V5 (ZBTB25)
250kDa
150kDa
100kDa
75kDa
50kDa
37kDa
25kDa
HA (CoV2 Mpro)
150kDa
100kDa
75kDa
50kDa
37kDa
25kDa
V5 (ZBTB25)

Fig. 3D

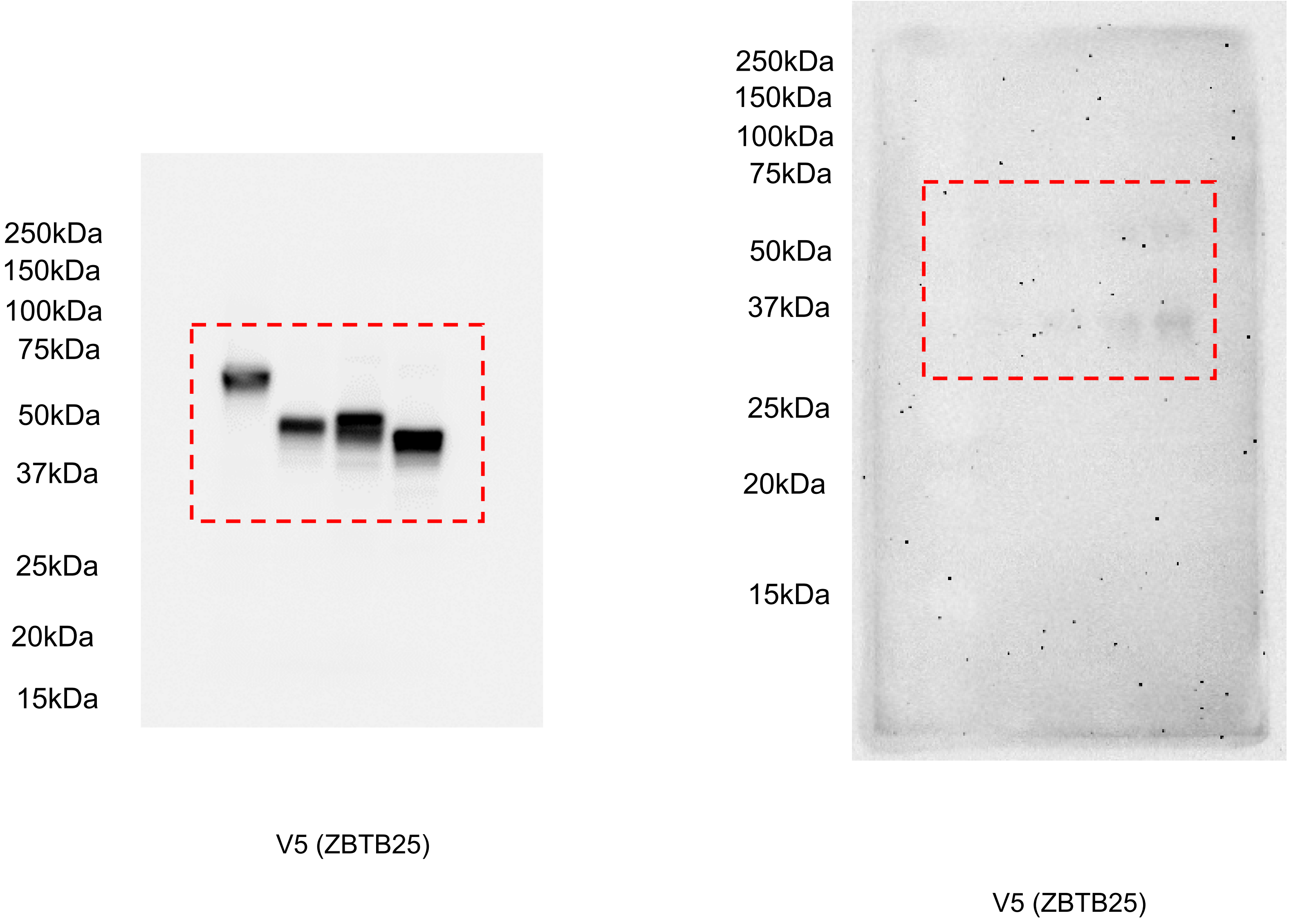
250kDa
150kDa
100kDa
75kDa
50kDa
37kDa
25kDa
20kDa
15kDa
V5 (ZBTB25)
250kDa
150kDa
100kDa
75kDa
50kDa
37kDa
25kDa
20kDa
15kDa
V5 (ZBTB25)

Fig. 3E

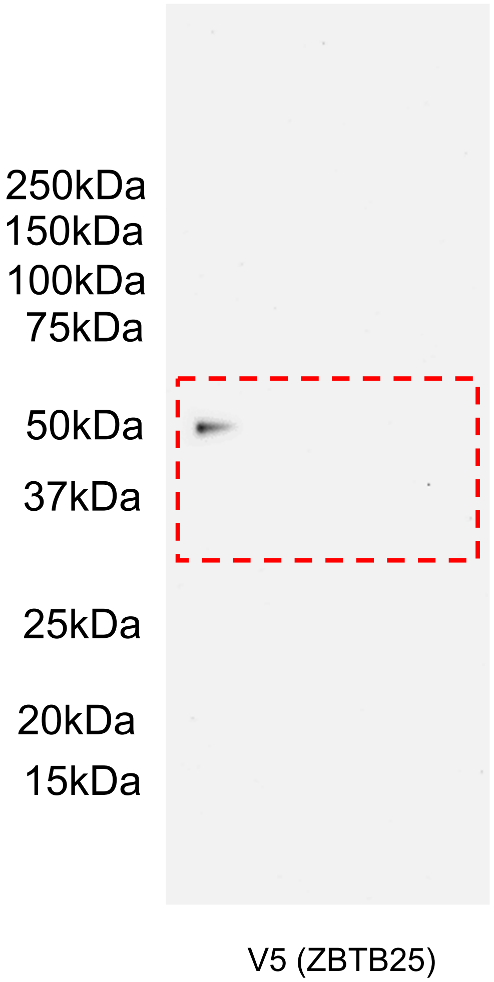
250kDa
150kDa
100kDa
75kDa
50kDa
37kDa
25kDa
20kDa
15kDa
V5 (ZBTB25)

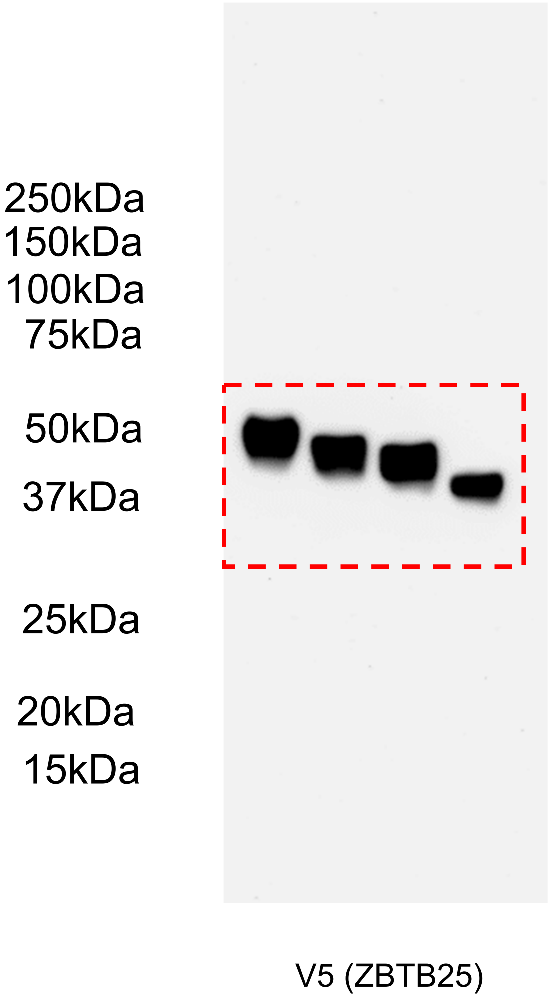
250kDa
150kDa
100kDa
75kDa
50kDa
37kDa
25kDa
20kDa
15kDa
V5 (ZBTB25)

Fig. 3F

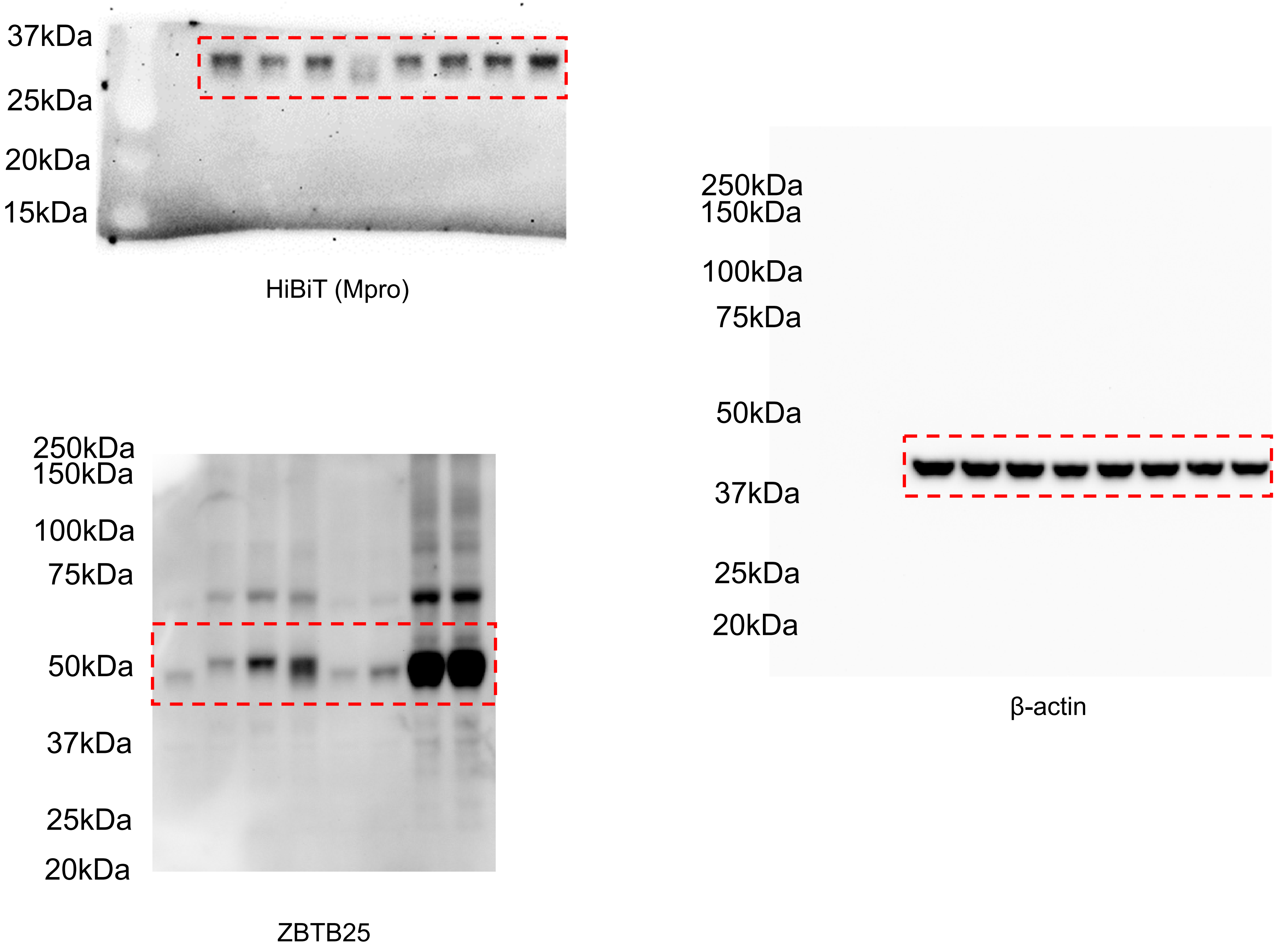
37kDa
25kDa
20kDa
15kDa
HiBiT (Mpro)
250kDa
150kDa
100kDa
75kDa
50kDa
37kDa
25kDa
20kDa
ZBTB25
250kDa
150kDa
100kDa
75kDa
50kDa
37kDa
25kDa
20kDa
β-actin

Fig. 3H

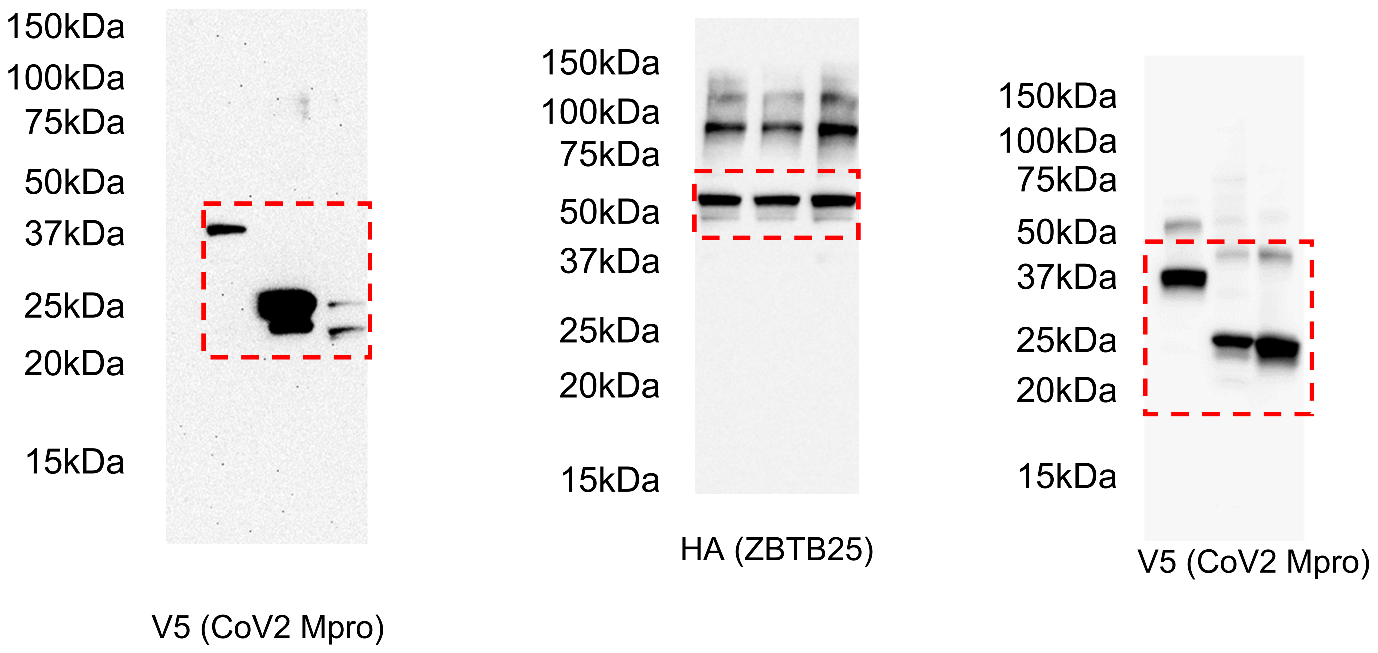
150kDa
100kDa
75kDa
50kDa
37kDa
25kDa
20kDa
15kDa
V5 (CoV2 Mpro)
150kDa
100kDa
75kDa
50kDa
37kDa
25kDa
20kDa
15kDa
HA (ZBTB25)
150kDa
100kDa
75kDa
50kDa
37kDa
25kDa
20kDa
15kDa
V5 (CoV2 Mpro)

Fig. 3I

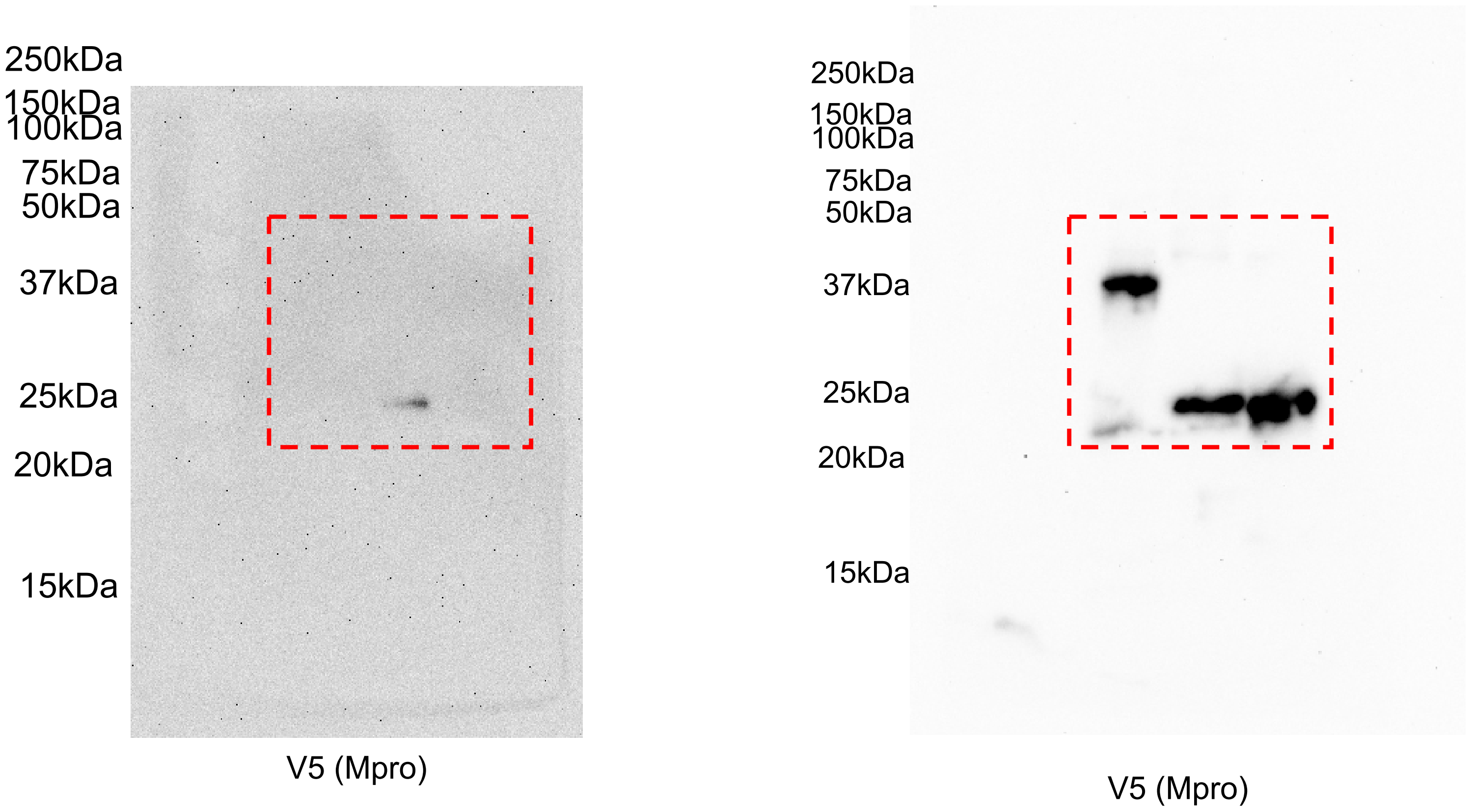

250kDa
150kDa
100kDa
75kDa
50kDa
37kDa
25kDa
20kDa
15kDa
V5 (Mpro)
250kDa
150kDa
100kDa
75kDa
50kDa
37kDa
25kDa
20kDa
15kDa
V5 (Mpro)

Fig. 4B

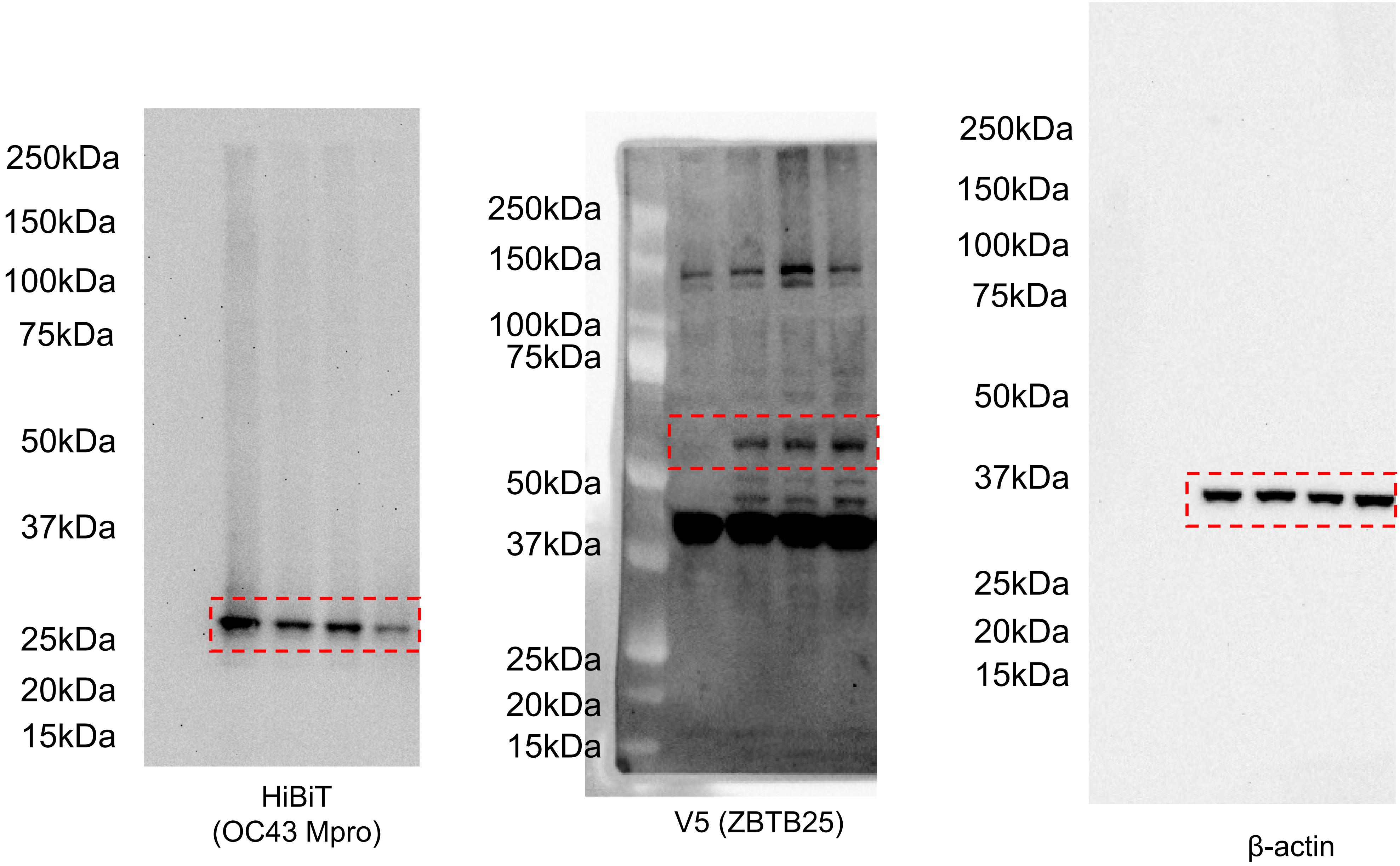
250kDa
150kDa
100kDa
75kDa
50kDa
37kDa
25kDa
20kDa
15kDa
HiBiT
(OC43 Mpro)
250kDa
150kDa
100kDa
75kDa
50kDa
37kDa
25kDa
20kDa
15kDa
V5 (ZBTB25)
250kDa
150kDa
100kDa
75kDa
50kDa
37kDa
25kDa
20kDa
15kDa
β-actin

Fig. 4E

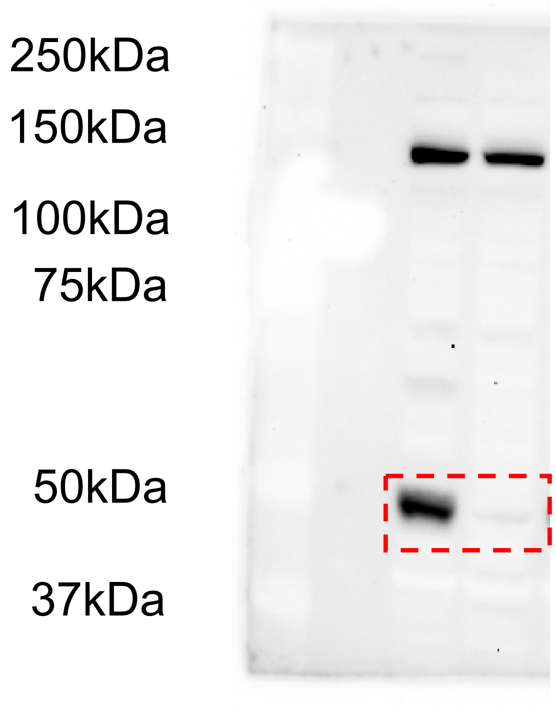

ZBTB25

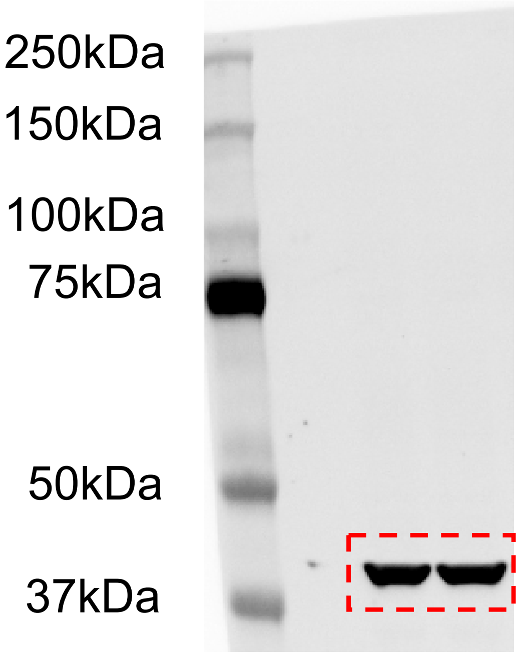

β-actin

Fig. 4H

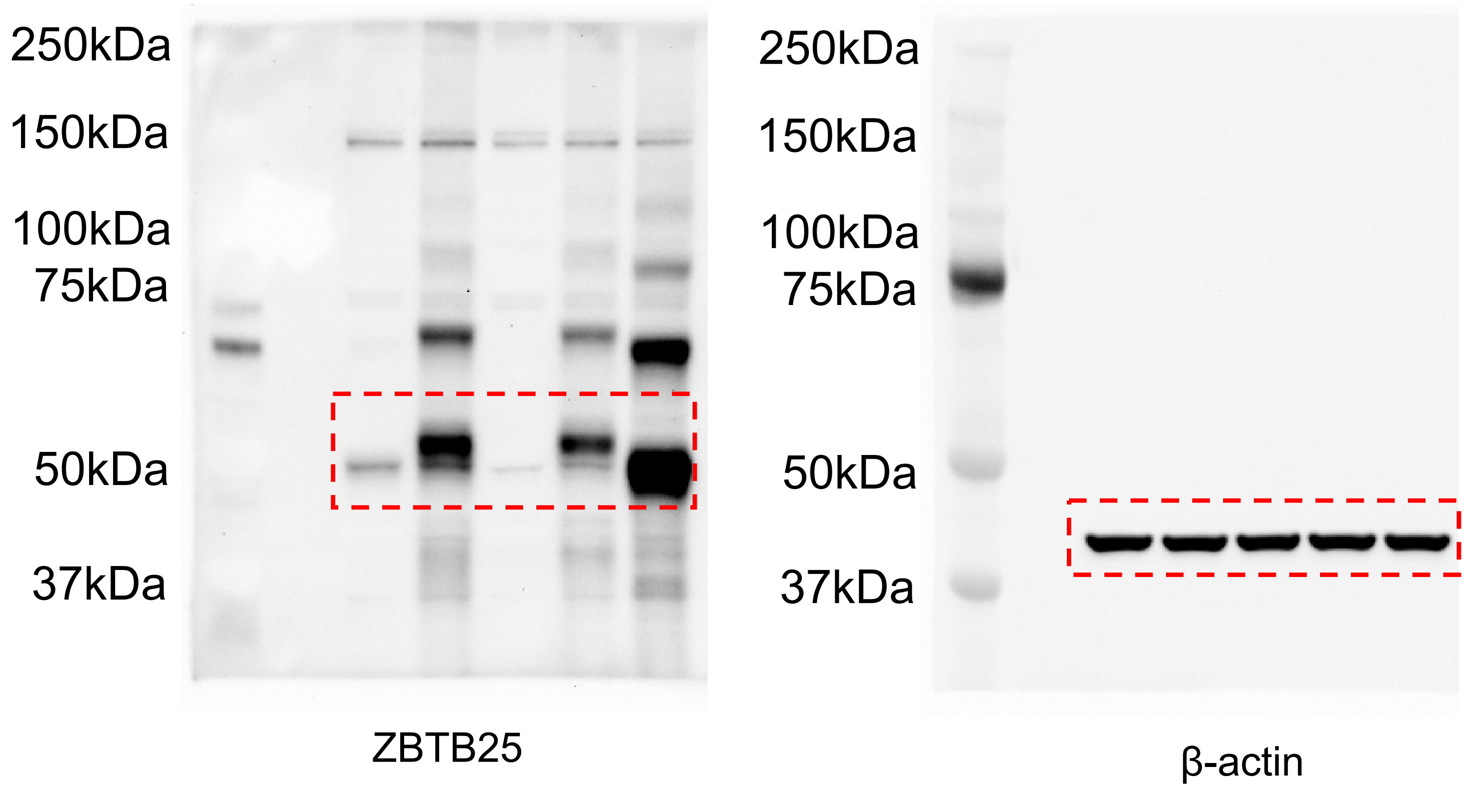
250kDa
150kDa
100kDa
75kDa
50kDa
37kDa
ZBTB25
250kDa
150kDa
100kDa
75kDa
50kDa
37kDa
β-actin

Fig. S1A

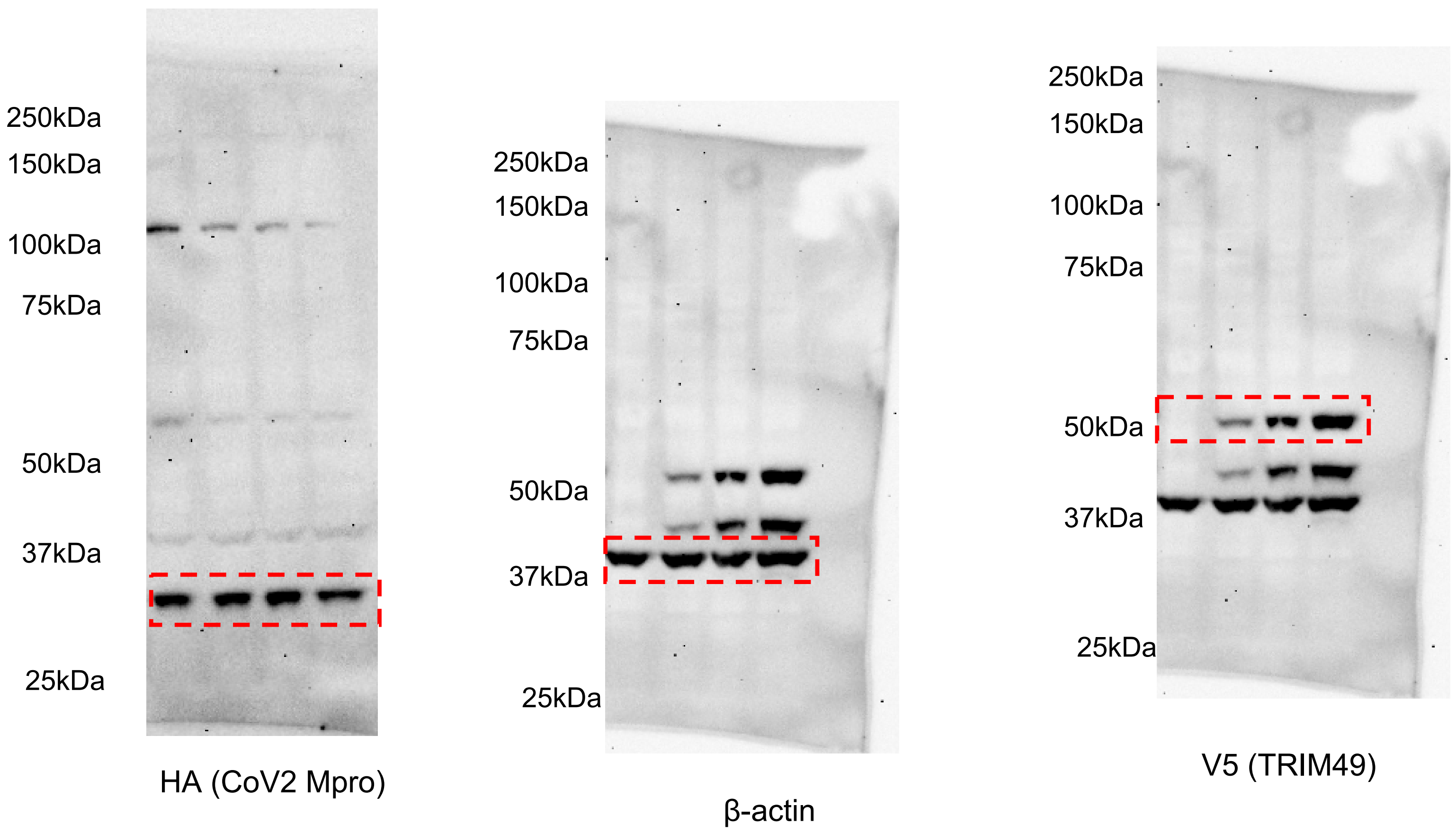
250kDa
150kDa
100kDa
75kDa
50kDa
37kDa
25kDa
HA (CoV2 Mpro)
250kDa
150kDa
100kDa
75kDa
50kDa
37kDa
25kDa
β-actin
250kDa
150kDa
100kDa
75kDa
50kDa
37kDa
25kDa
V5 (TRIM49)

Fig. S1B

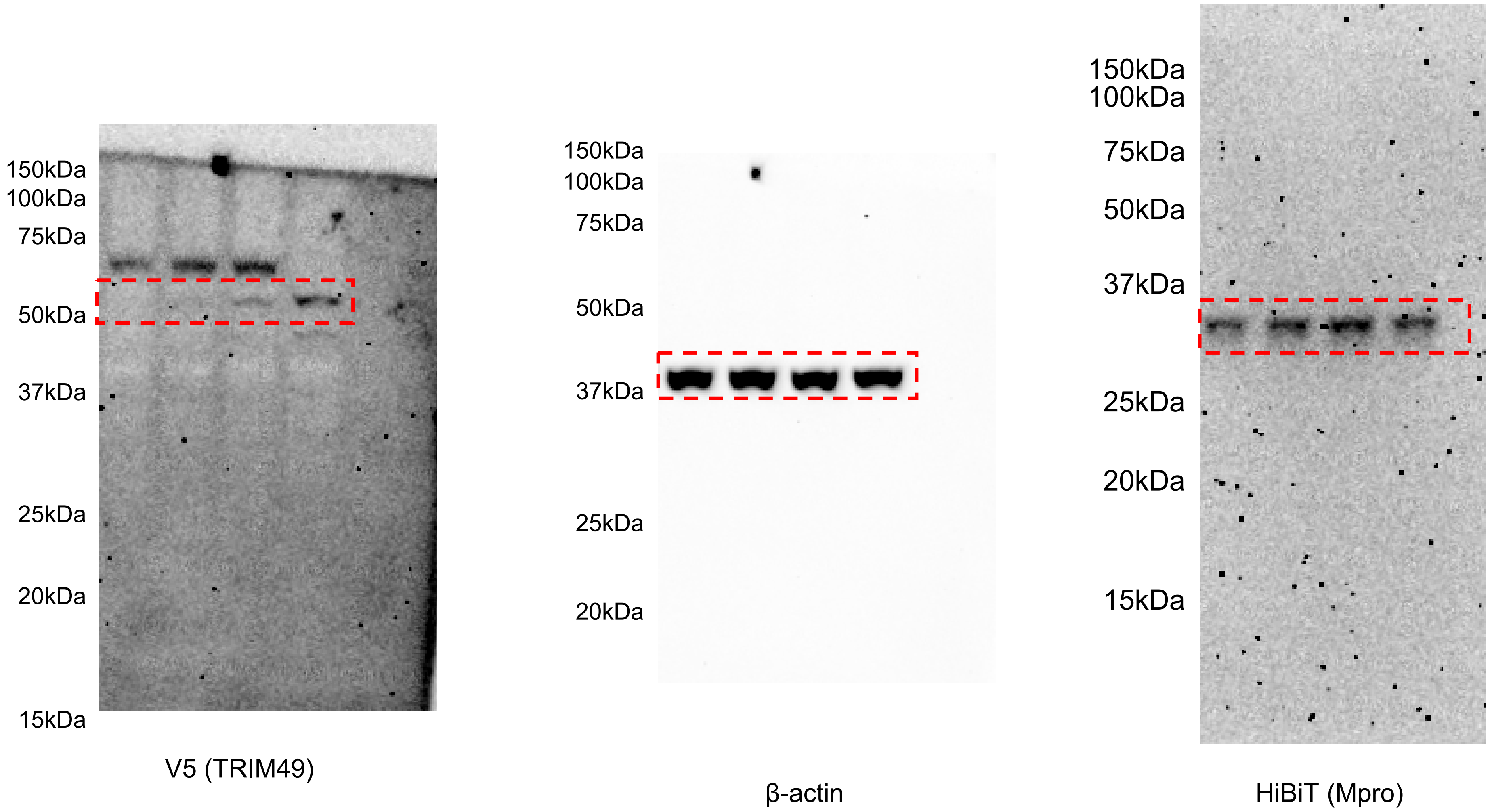
150kDa
100kDa
75kDa
50kDa
37kDa
25kDa
20kDa
15kDa
V5 (TRIM49)
150kDa
100kDa
75kDa
50kDa
37kDa
25kDa
20kDa
β-actin
150kDa
100kDa
75kDa
50kDa
37kDa
25kDa
20kDa
15kDa
HiBiT (Mpro)
